# Cardiovascular effects of *Matricaria chamomilla* extract: calcium channel modulation and vasorelaxant activity

**DOI:** 10.1007/s00114-025-02065-0

**Published:** 2026-01-19

**Authors:** Omonturdiev Sirojiddin, Abdullaev Izzatullo, Gayibov Ulugbek, Inomjonov Dolimjon, Gayibova Sabina, Makhmudov Rustamjon, Aripov Takhir, Emine İncilay Torunoğlu, Erdi Can Aytar

**Affiliations:** 1 Institute of Bioorganic Chemistry named after A.Sadykov, Laboratory of Plant Cytoprotectors, Mirzo Ulugbek, 83, Tashkent, Uzbekistan; 2 Namangan State University, Namangan, Uzbekistan; 3 Faculty of Medicine, Department of Medical Biochemistry, Necmettin Erbakan University, Konya, Türkiye 42090 Turkey; 4 Faculty of Agriculture Department of Horticulture, Usak University, Uşak, Türkiye 64200 Turkey

**Keywords:** Calcium ion channels, Cardiovascular system, Hypertension, Matricaria *Chamomilla*, Molecular docking

## Abstract

This study aimed to investigate the vasorelaxant potential of *M. chamomilla* extract and its modulatory effects on calcium ion channels.In vitro experiments assessed the extract’s impact on voltage-gated and GPCR-mediated calcium channels in aortic preparations. In vivo, the Tail Cuff method evaluated blood pressure-lowering effects in adrenaline-induced hypertensive rats. Phytochemical profiling was performed via GC-MS, and molecular docking assessed interactions of key compounds with vascular regulation targets (7VFS, 8THK, 3NOS). In vitro, 5 µg/ mL of the extract slightly increased aortic contractility (3.9 ± 3.4%), whereas 60 µg/ mL markedly reduced it (89.5 ± 3.1%). At 50 µg/ mL, it inhibited phenylephrine-induced GPCR-mediated contractions by 84.9 ± 3.8%. In vivo, 40 mg/kg of the extract lowered systolic and diastolic pressures to 150 mmHg and 110 mmHg, respectively. GC-MS identified pinocarveol, coumarin, apigenin derivatives, and dicaffeoylquinic acids. Molecular docking revealed strong affinities of apigenin-7-O-neohesperidoside and other compounds to key vascular targets. Both experimental approaches consistently demonstrated vasorelaxant activity, likely linked to polyphenol and flavonoid content. *M. chamomilla* extract exhibits significant vasorelaxant and antihypertensive effects, mediated through modulation of calcium channels and bioactive polyphenols. These findings support its potential as a therapeutic agent for hypertension and hypoxia-related cardiovascular disorders, warranting further clinical investigation.

## Introduction

Medicinal plants have long been recognized as valuable sources of bioactive compounds with therapeutic potential and continue to serve as important reservoirs for drug discovery (Atanasov et al. 2015). Traditionally used in Indian Ayurveda, as well as in Chinese, African, and Mediterranean herbal medicine systems, plant-derived products remain a primary treatment option for many populations due to their bioavailability and affordability. Numerous natural compounds have demonstrated significant biological activities and drug-like properties, making them effective against various human diseases, including cancer (Amin et al. 2021; Murali et al. 2021), gastrointestinal disorders(Ashktorab et al. 2019), infectious diseases (Mu et al. 2021), and cardiovascular conditions(Shaito et al. 2020).

*Matricaria chamomilla* (commonly known as chamomile) is a well-known medicinal plant belonging to the Asteraceae family. It is an annual, cold-resistant herb that can grow in all soil types (Lim 2014). Native to southern and eastern Europe as well as northern and western Asia, it is now widely cultivated across many regions worldwide (Singh et al. 2011). Traditionally, *M. chamomilla* has been used for the treatment of various ailments, including gastrointestinal disorders (Menale et al. 2022), common cold(Güzel et al. 2015), liver diseases(Živković et al. 2020), and neuropsychiatric and respiratory conditions (Neves et al. 2009). Additionally, due to its analgesic and anti-infective properties, it is commonly employed against pain and infections, and is also used in the treatment of skin, eye, and oral diseases(Petrakou et al. 2020).

Plant secondary metabolites have shown significant effects in the treatment of cardiovascular diseases at the cellular level, making this research highly relevant to modern medicine and pharmacology (Khazdair M et al., 2022, Abdullaev et al. 2024). Smooth muscle contraction is closely associated with the increase in Ca^2+^ ion levels in the cytosol. These ions are regulated through two primary mechanisms. First, during membrane depolarization, ions enter the cell via L-type Ca^2+^ channels. Second, Ca^2+^ ions are released from the sarcoplasmic reticulum (SR) via ryanodine and IP3 receptors. The processes of membrane depolarization and repolarization are essential in smooth muscle cell activity, as they modulate ion transport. Humoral factors, hormones, and mechanical influences, such as cell relaxation, can significantly affect membrane reactions and ion transport (Ghosh et al. 2017).

Considering these issues, this study investigates the mechanisms through which *M. chamomilla* extract affects the ion transport systems of rat aortic smooth muscle. Literature analysis has confirmed that certain extracts exhibit relaxant effects (Zoirovich et al. 2024). Screening drugs through both in vivo and in vitro experiments can provide a comprehensive understanding of their mechanisms of action. In this study, we investigated the effects of *M. chamomilla* extract through both in vitro and in vivo experiments(Awaad et al. 2018).

The study of the mechanisms of biologically active substances is essential for the development of pharmaceutical agents with antioxidant, relaxant, antiviral, antibacterial, and other pharmacological activities.

## Materials

### Plant material and extraction

The *M. chamomilla*, extract used in this study was supplied by “Bioton” LTD, a company based in Tashkent, Uzbekistan. *M. chamomilla* is a medicinal plant widely prevalent in Uzbekistan *M. chamomilla* extract was obtained using ethanol as the extraction solvent, and the ethanol was subsequently evaporated using a rotary evaporator. The resulting ethanol extract was concentrated by evaporating the solvent under reduced pressure at 40 °C using a rotary evaporator. The concentrated extract was then transferred into airtight containers and stored at 4 °C for further analyses (Azimova et al. 2024; Gaibullayeva et al. 2024).

### Aortic tissue preparation

All experimental procedures and preoperative care protocols were approved by the institutional Animal Ethics Committee. Animals were maintained under standard vivarium conditions (humidity: 55%–65%, temperature: 22 ± 2 °C) with ad libitum access to drinking water and standard laboratory chow. All procedures were conducted in accordance with the European Directive 2010/63/EU for the protection of animals used for scientific purposes. The study protocol was approved by the Animal Ethical Committee of the Institute of Bioorganic Chemistry, AS RUz (Protocol No: 133/1a/h; approved on August 4, 2014).

Surgical procedures were performed under sodium pentobarbital anesthesia and all efforts were made to minimize animal suffering. Aortic tissues were obtained from white male rats weighing 200–250 g(Zaripova et al. 2024). Animals were euthanized via cervical dislocation, after which the thoracic cavity was opened, and the aorta was carefully excised. The isolated aorta was immediately placed in a 5 mL organ bath containing Krebs-Henseleit physiological solution composed of (in mM): NaCl 120.4, KCl 5, NaHCO₃ 15.5, NaH₂PO₄ 1.2, MgCl₂ 1.2, CaCl₂ 2.5, glucose (C₆H₁₂O₆) 11.5, and HEPES (pH 7.4).

In the experiments a Ca²⁺-free Krebs solution supplemented with 1 mM EGTA was used. All physiological solutions were continuously oxygenated with carbogen (95% O₂, 5% CO₂) and maintained at 37 °C using a Water Bath, Daihan ultrathermostat. After removing connective tissue and fat from the aorta, the tissue was cut into 3–4 mm rings for subsequent experimental use (Zoirovich et al. 2024).

### Aortic-ring contraction studies

**Fig. 1:**
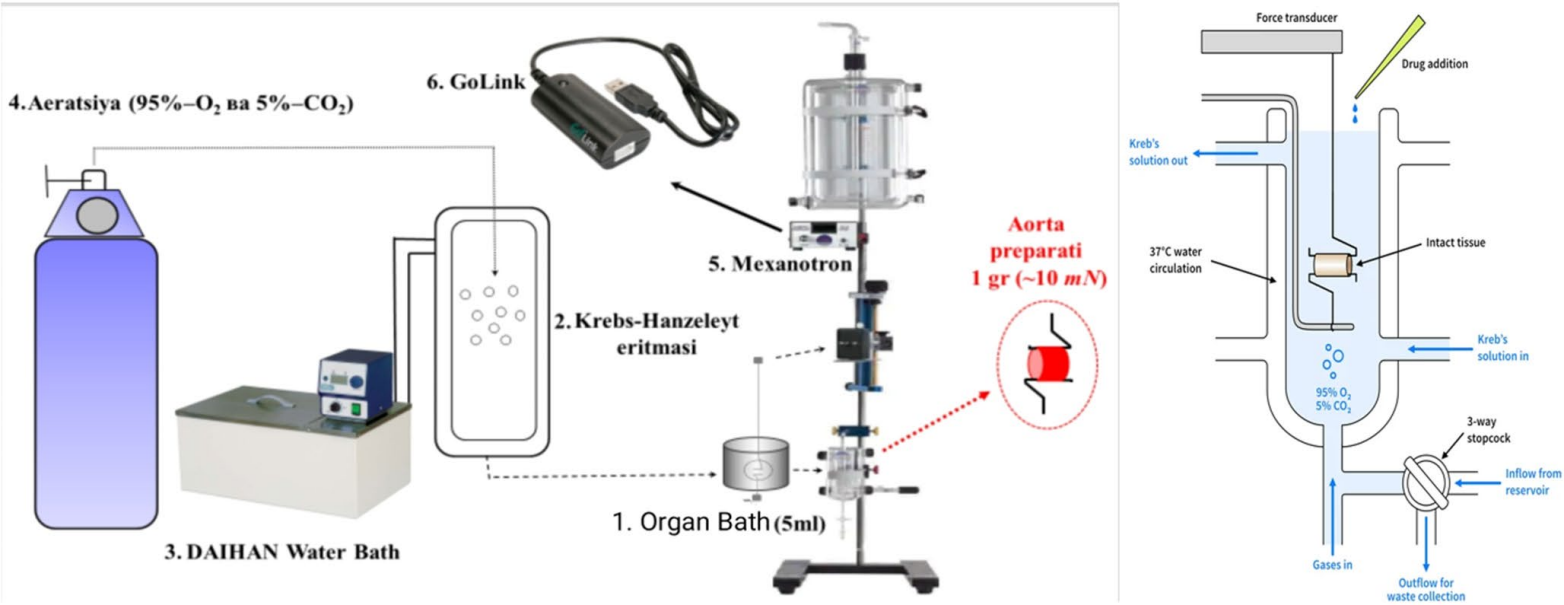
A schematic diagram of the setup used to control and measure the isometric contraction of isolated rat aortic smooth muscle is shown. (1) The organ bath (5 mL) is connected to a dedicated reservoir that circulates the solution. (2) The Krebs-Henseleit solution is used to maintain physiological conditions. (3) A thermostat regulates the temperature to ensure it remains constant and within the physiological range. (4) The system is continuously aerated with a gas mixture of 5% CO_2_ and 95% O2. The contractile activity of the aortic tissue is maintained within the experimental chamber. (5) An isometric transducer (Grass Instrument, USA) measures the contraction, and (6) The GoLink devices amplify the signal and assist in data collection (Vandier et al., 2002)

Aortic rings were mounted on a Radnoti isometric transducer system (USA) using platinum wire hooks. The tissues were allowed to equilibrate for 60 min under these conditions. An initial resting tension of 1 g (10 mN) was applied to each ring. The contractile force was transmitted from the transducer to a signal amplifier and subsequently recorded on a computer using a Go-link automated digital converter. The acquired data were analyzed statistically using specialized software packages (Fig. 1) (Mirzayeva et al. 2024).

### “Tail Cuff” method and blood pressure measurement

Arterial blood pressure in rats was measured using the tail cuff method, a widely employed non-invasive technique for assessing hemodynamic parameters in small animals. The procedure was conducted at the “BFM Pharmacology and Screening Laboratory” and the “Plant Cytoprotectors Laboratory” of the A. Sadykov Institute of Bioorganic Chemistry.

Blood pressure measurements were performed using the “Sistola” experimental device (Neurobotics, RF) in conjunction with the specialized software “AcqKnowledge 4.2 for MP150.” During the procedure, rats were gently restrained, and a cuff was placed around the tail artery. The cuff was inflated to temporarily occlude blood flow, then gradually deflated while monitoring the return of blood flow to determine systolic and diastolic pressure values.

All measurements were conducted in strict accordance with ethical guidelines to minimize stress and discomfort to the animals (Ibrahim et al. 2006).

### Experimental hypertension induction and medicinal plant extract treatment

Experimental hypertension was induced in rats by intraperitoneal injection of adrenaline hydrochloride (0.25 mg/kg). This procedure produced a stable hypertensive response, which was monitored for 180 min following the injection.

Subsequently, medicinal plant extracts were administered intraperitoneally to experimental groups at doses of 40, 70, and 100 mg/kg. Blood pressure measurements were recorded at specific time intervals throughout the experiment to evaluate the effects of the plant extracts on the induced hypertensive state.

**Fig. 2:**
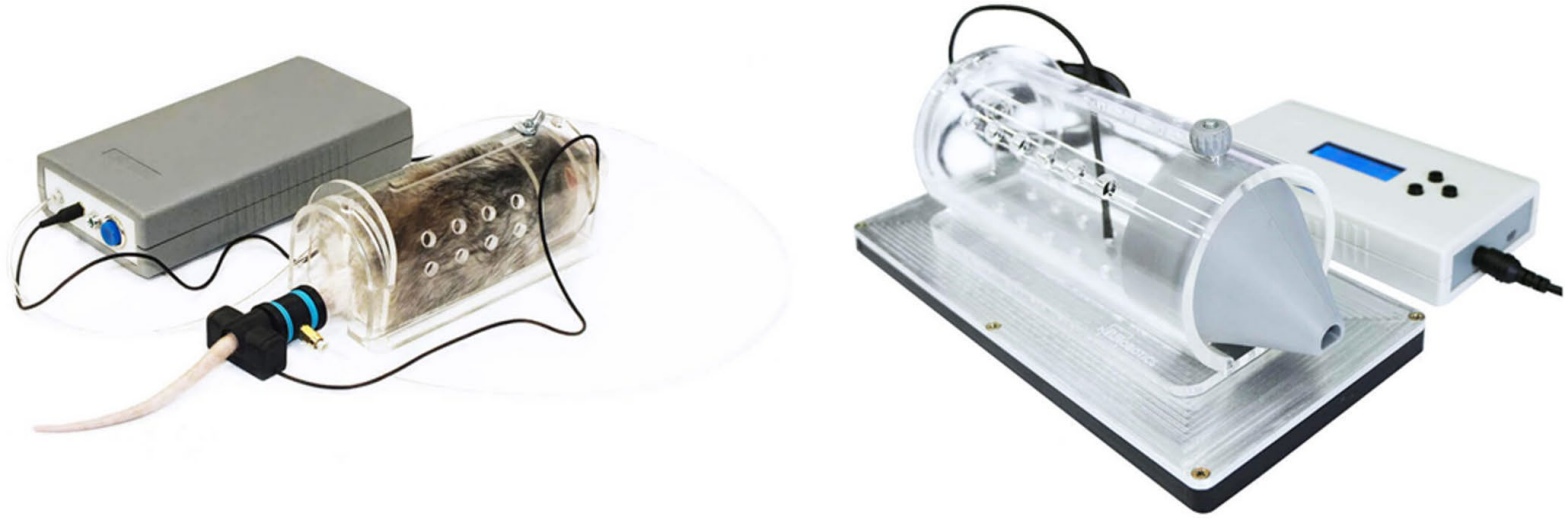
Experimental device “Sistola” (Neurobotics, RF) for noninvasive recording of arterial blood pressure in the blood vessel of the tail artery in rats

The data were analyzed using the “AcqKnowledge 4.2 for MP150” software to determine the impact of the medicinal plant extracts on arterial blood pressure under hypertensive conditions (Fig. 2) (Borkowski and Quinn 1985).

### ESI–QTOF-MS/MS analysis of polyphenols with gradient elution

Mass spectrometric studies of the isolated polyphenols were conducted using a Q-TOF LC-MS Agilent Technologies 6520B instrument under the following conditions: ionization source – ESI, drying gas flow – 5 L/min, drying gas temperature – 300 °C, voltage on: skimmer cone – 20 V, fragmentor 125 V, mass range: in MS mode 100–2000 m/z, and in Targeted MS/MS mode 50–2000 m/z, collision energy – 35, 50 eV. Ionization method: negative. Samples were introduced into the mass spectrometer using an Agilent Technologies 1200 series chromatograph, Zorbax SBC18 column, 3 μm, 0.5 × 150 mm. Mobile phase: A – 0.1% formic acid solution, B – acetonitrile + 0.1% formic acid. Elution was performed on an Agilent Technologies 1260 Cap Pump instrument at a flow rate of 15 µL/min. Gradient concentration of solution B – in minutes: 0–5 min – 20%, 20 min – 25%, 25 min – 30%, 25.1–30 min – 60%, 35 min – 20%. Solutions were degassed using an Agilent Technologies 1260 µ-degasser. Samples were applied to the column using an Agilent Technologies Micro WPS device at 1 µL from a polyphenol solution with a concentration of 0.1 mg/mL.

For compound identification, they were preliminarily characterized using MS data, together with interpretation of MS/MS spectra, by comparison with those found in the literature. During the identification process, the following public databases were studied: Chemical Entities of Biological Interest (ChEBI, https://www.ebi.ac.uk/chebi/), Chemical Compounds Deep Data Source (https://www.molinstincts.com/), ChemSpider (www.chemspider.com) and Phenol_Explorer (www.phenol-explorer.eu).

### Phytochemical screening and molecular Docking approach

Based on the in vitro and in vivo findings of this study, *M. chamomilla* extract demonstrated significant vasorelaxant effects through multiple mechanisms in vascular smooth muscle. The extract markedly inhibited contractions induced by high potassium (KCl, 50 mM), suggesting a blockade of calcium influx via voltage-dependent L-type calcium channels. To investigate this mechanism at the molecular level, the PDB structure 7VFS, representing the L-type calcium channel (Cav1.1) in complex with the known blocker verapamil, was selected. This model provides a suitable conformation to explore potential channel-binding interactions of the extract. Additionally, the extract significantly suppressed phenylephrine-induced contractions mediated by α1-adrenergic receptors. Since this response mimicked the pharmacological effects of phentolamine, a non-selective α-blocker, the 8THK structure—an active conformation of the human α1A-adrenergic receptor bound to a selective agonist and complexed with the Gq protein—was chosen. This model enables the exploration of potential antagonistic interactions of the extract with GPCR-mediated pathways. Moreover, endothelium-dependent mechanisms were implicated by the observed attenuation of the extract’s effects in the presence of L-NAME, an eNOS inhibitor, and following endothelial denudation. These findings suggested that nitric oxide (NO) signaling contributes to the extract’s vasorelaxant action. Therefore, the 3NOS structure, depicting human endothelial nitric oxide synthase (eNOS) bound to its substrate L-arginine, was selected to explore how the extract may influence NO production. Collectively, the selection of these three PDB structures enables a comprehensive molecular docking analysis of the extract’s effects on voltage-gated calcium entry, GPCR-mediated contraction, and NO-dependent endothelial relaxation—mechanisms all supported by the experimental outcomes of this study.

The principal bioactive compounds identified from plant extracts were retrieved from the PubChem database in Structure Data File (SDF) format. These molecular files were subsequently converted into Protein Data Bank (PDB) format using Discovery Studio Visualizer to prepare them for molecular docking procedures. To account for molecular flexibility, rotatable bonds were defined, and torsional parameters refined. The processed ligand structures were then imported into PyRx, where they were transformed into PDBQT format using the integrated AutoDock Vina plugin.

Three target proteins relevant to the study were selected from the Protein Data Bank (https://www.rcsb.org). Prior to docking, protein structures underwent standard preparation steps: all crystallographic water molecules were removed, any non-standard amino acid residues were corrected, and Gasteiger partial charges were assigned using AutoDock Tools (ADT) within the AutoDock 4.2 suite. The prepared protein structures were then exported in PDBQT format for docking compatibility.

Molecular docking simulations were carried out using AutoDock Vina. Ligands were subjected to geometry optimization while preserving their torsional flexibility. Appropriate atomic charges were calculated and assigned. For each ligand, multiple binding conformations were generated; the pose with the lowest binding energy was considered the most favorable. Resulting protein–ligand complexes were examined using Discovery Studio Visualizer to characterize key molecular interactions, including hydrogen bonding, hydrophobic effects, and other non-covalent stabilizing forces.

### Statistical analysis and illustration

All experimental data were analyzed using statistical methods to ensure the reliability and significance of the results. The data were expressed as mean ± standard error of the mean (SEM). The Student’s t-test was used to compare the differences between two groups, and statistical significance was considered at *p* < 0.05 and *p* < 0.01. All statistical analyses were carried out using OriginLab OriginPro v.8.5 SR1 (EULA, Northampton, MA 01060–4401, USA). The results were considered statistically significant when p values were less than 0.05.

## Results

### Study of *M. chamomilla *extract on voltage-dependent Ca²⁺ Ion Channels: Effects with verapamil blocker and varying Ca²⁺ concentrations

**Fig. 3:**
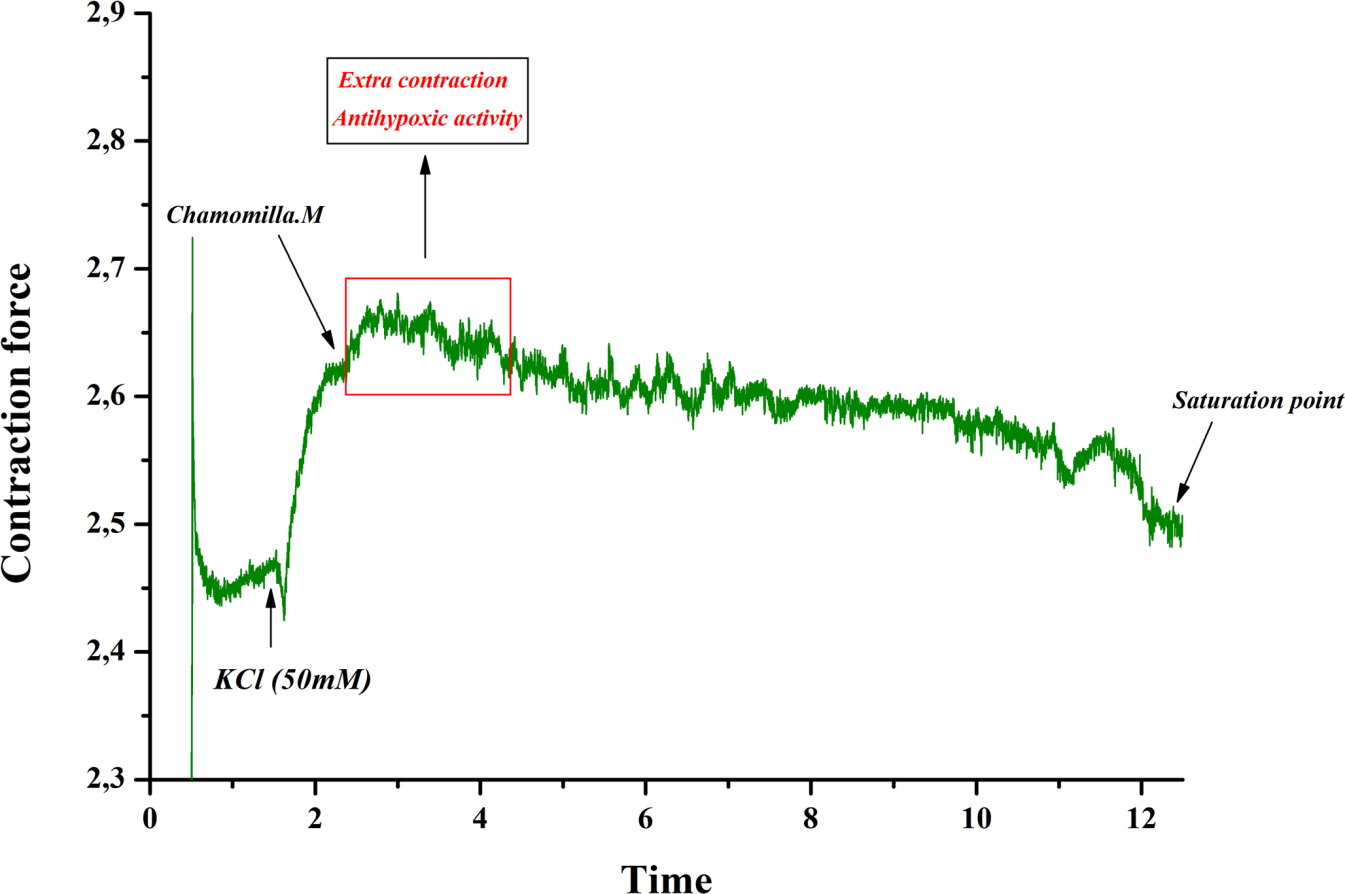
Original Recording Enhanced with OriginPro 8.5: Dose-Dependent Effect of *M. chamomilla* M on KCl-Induced Contraction

**Fig. 4:**
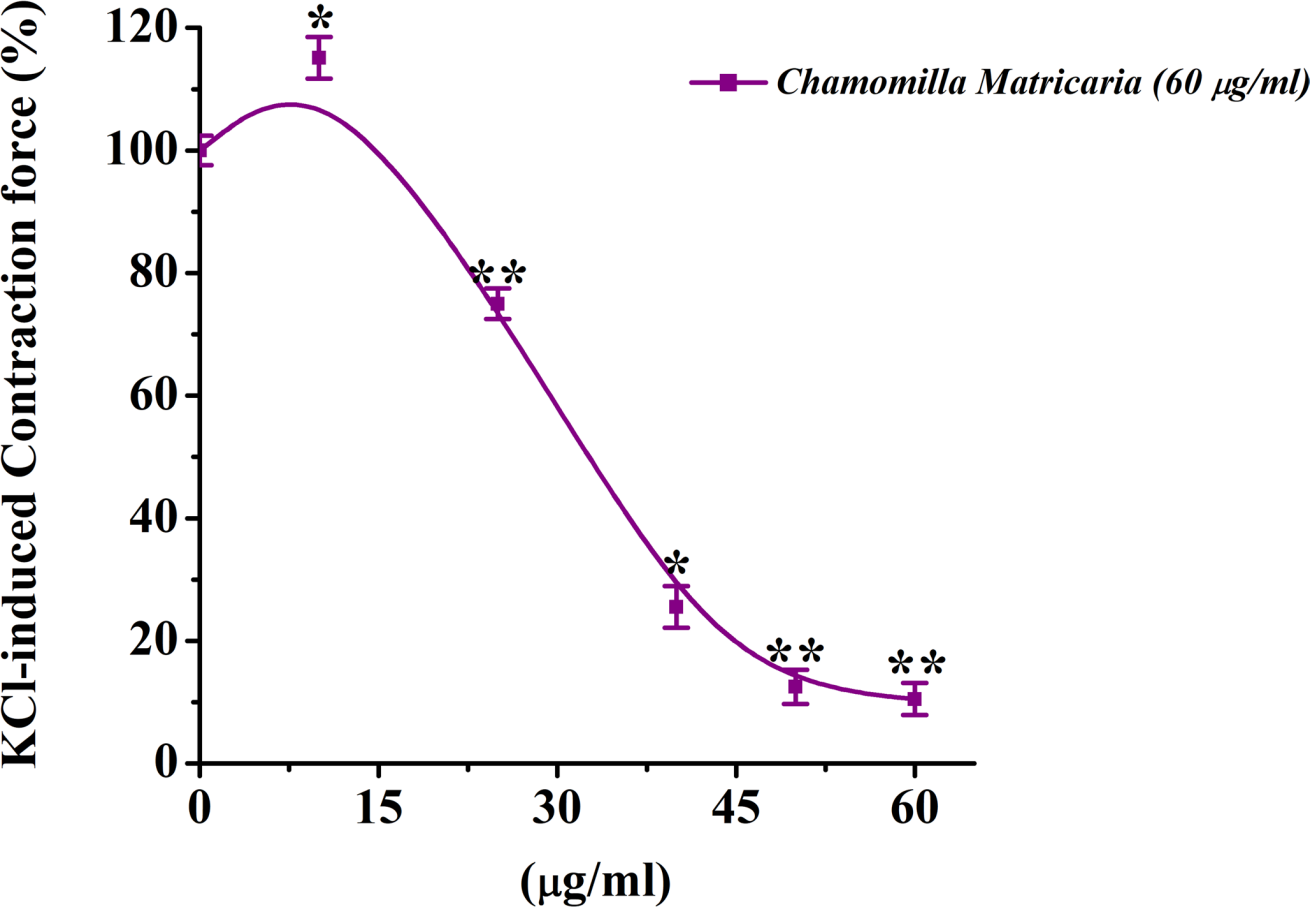
Effect of *M. chamomilla* extract on the contraction of rat aortic smooth muscle preparations induced by KCl (50 mM). The ordinate axis represents the contraction force of the aortic preparation induced by KCl (50 mM), with 100% set as the baseline. The abscissa axis represents the concentration of *M. chamomilla* extract (µg/mL). Statistical significance is indicated as *p < 0.05, **p < 0.01; *n* = 5–6)

The effect of *M. chamomilla* extract on contractions of rat aortic preparations induced by KCl (50 mM) was investigated. The extract showed a concentration-dependent relaxant effect (10–60 µg/mL). Specifically, at 10 µg/mL, the contractile activity of the aortic preparation increased by 3.9 ± 3.4% compared to the control, while at 60 µg/mL, it decreased by 89.5 ± 3.1% (Figs. 3 and 4).

**Fig. 5:**
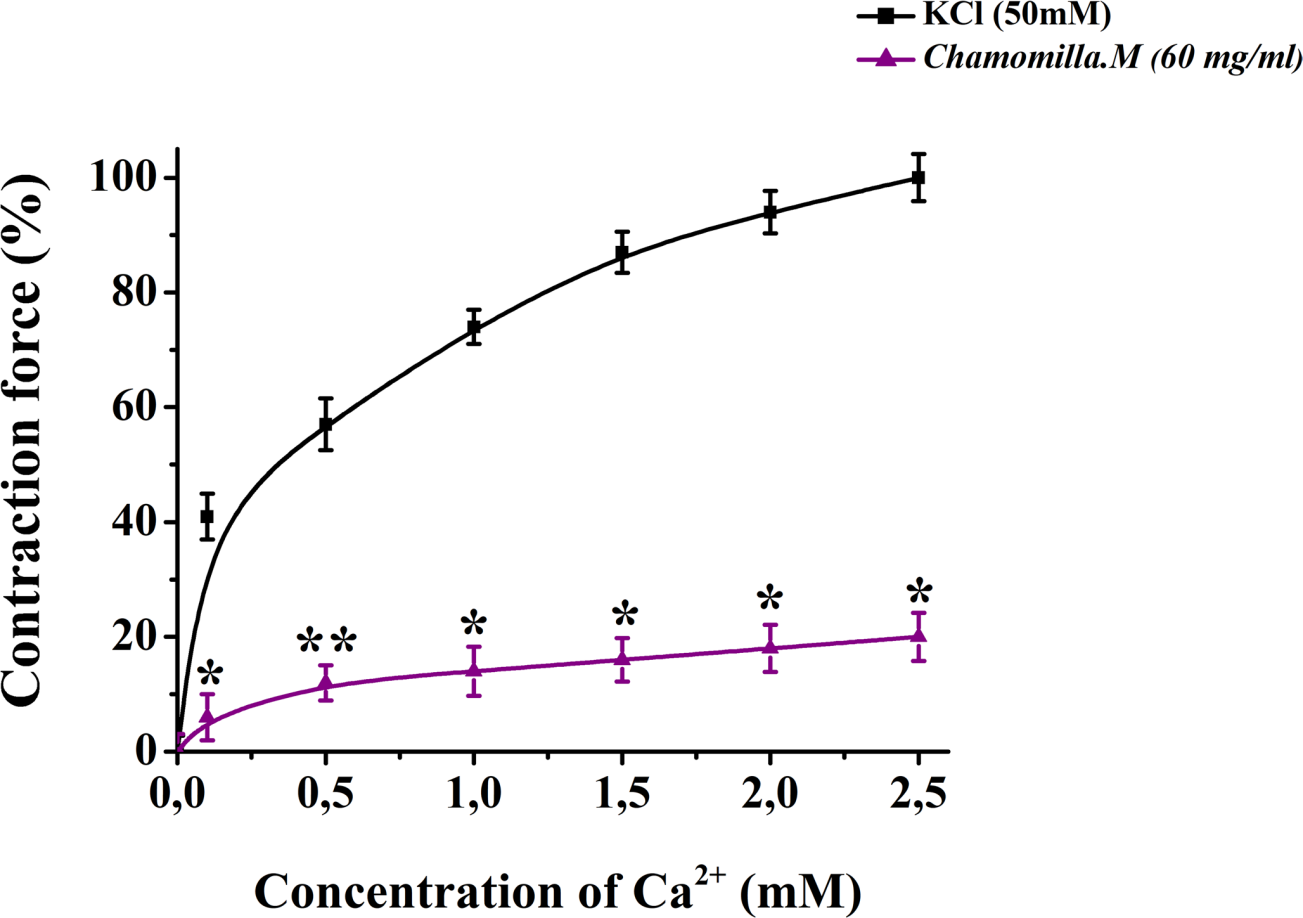
Effect of *M. chamomilla* extract on the relaxation activity in response to [Ca2+] concentration in the environment. The ordinate axis represents the contraction force of the aortic preparation induced by KCl (50 mM), with 100% set as the baseline. The abscissa axis represents the Ca2 + concentration (0–2.5 mM). Statistical significance is indicated as *p < 0.05, **p < 0.01; *n* = 4–5)

Based on the experimental results, chamomile extract exhibited a significant relaxant effect on contractions of aortic preparations induced by KCl (50 mM). Dose-dependent contractions were observed in aortic preparations when the Ca²⁺ ion concentration in Krebs solution containing 50 mM KCl was varied. Under these conditions, *M. chamomilla* extract significantly reduced aortic contraction compared to the control, indicating its effect on Ca²⁺ ion influx (Fig. 5).

**Fig. 6:**
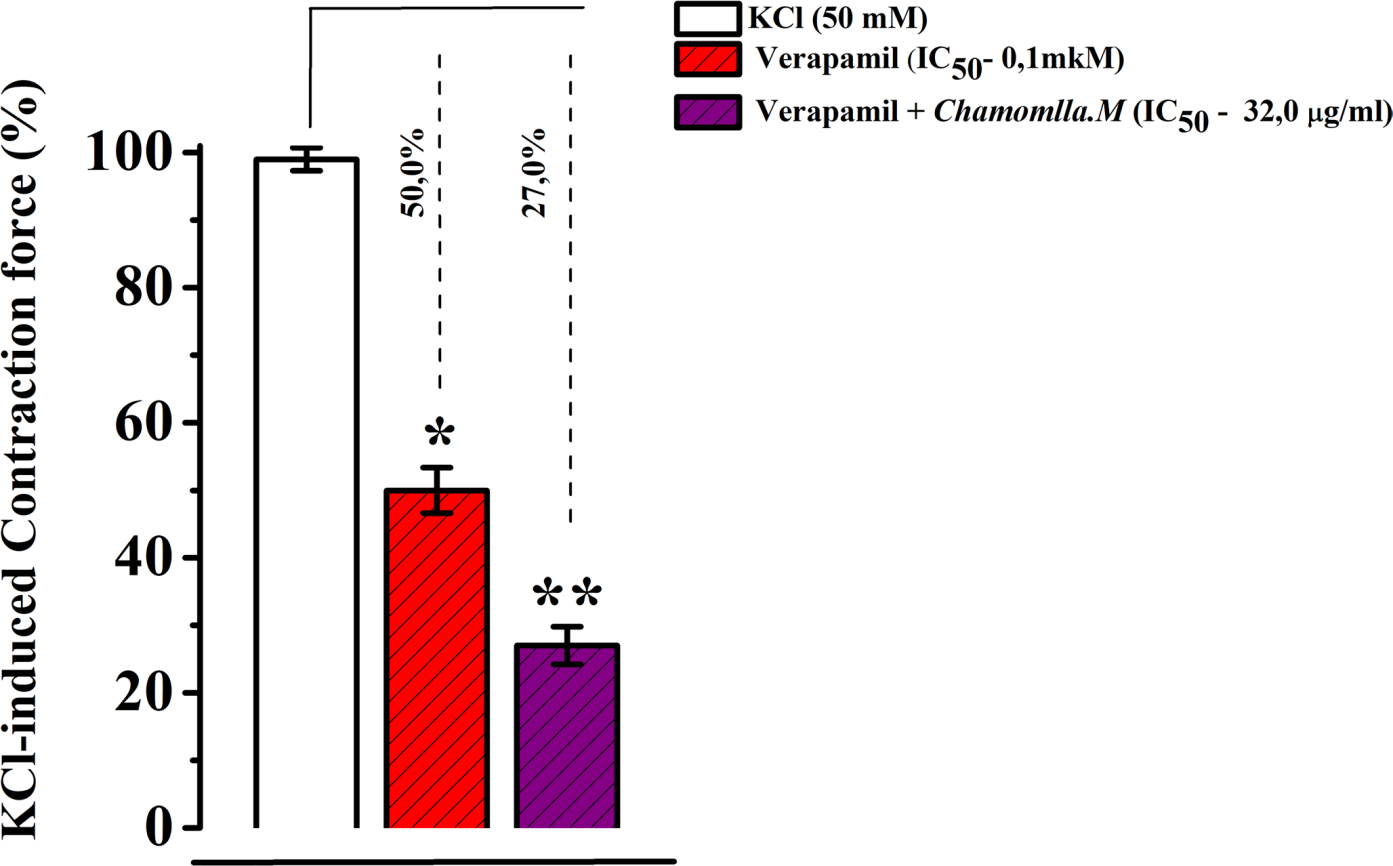
Interaction between *M. chamomilla* extract and the voltage dependent Ca^2+^ channel blocker verapamil (EC50) on the contraction of aortic preparations induced by KCl (50 mM). The ordinate axis represents the contraction force of the aortic smooth muscle preparation induced by KCl (50 mM), with 100% set as the baseline. Statistical significance is indicated as *p < 0.05, **p < 0.01; *n* = 5–7)

The experimental results indicate that the tested extract effectively inhibits Ca²⁺ ion entry through voltage-dependent Ca²⁺ channels in the cell membrane, thereby producing a relaxant effect on contractions induced by KCl. A verapamil concentration (0.1 µM) that produces half-maximal contraction in aortic preparations induced by KCl (50 mM) was used. When comparing the effects of verapamil (0.1 µM) and chamomile extract (EC₅₀), it was observed that the extract further reduced the amplitude of aortic contraction by 23 ± 3.6% compared to the control during incubation (Fig. 6).

### Studying of *M. chamomilla* extract on GPCR-mediated Ca²⁺ ion channels: Effects with phentolamine blocker and Ryr, IP3R pathways

**Fig. 7:**
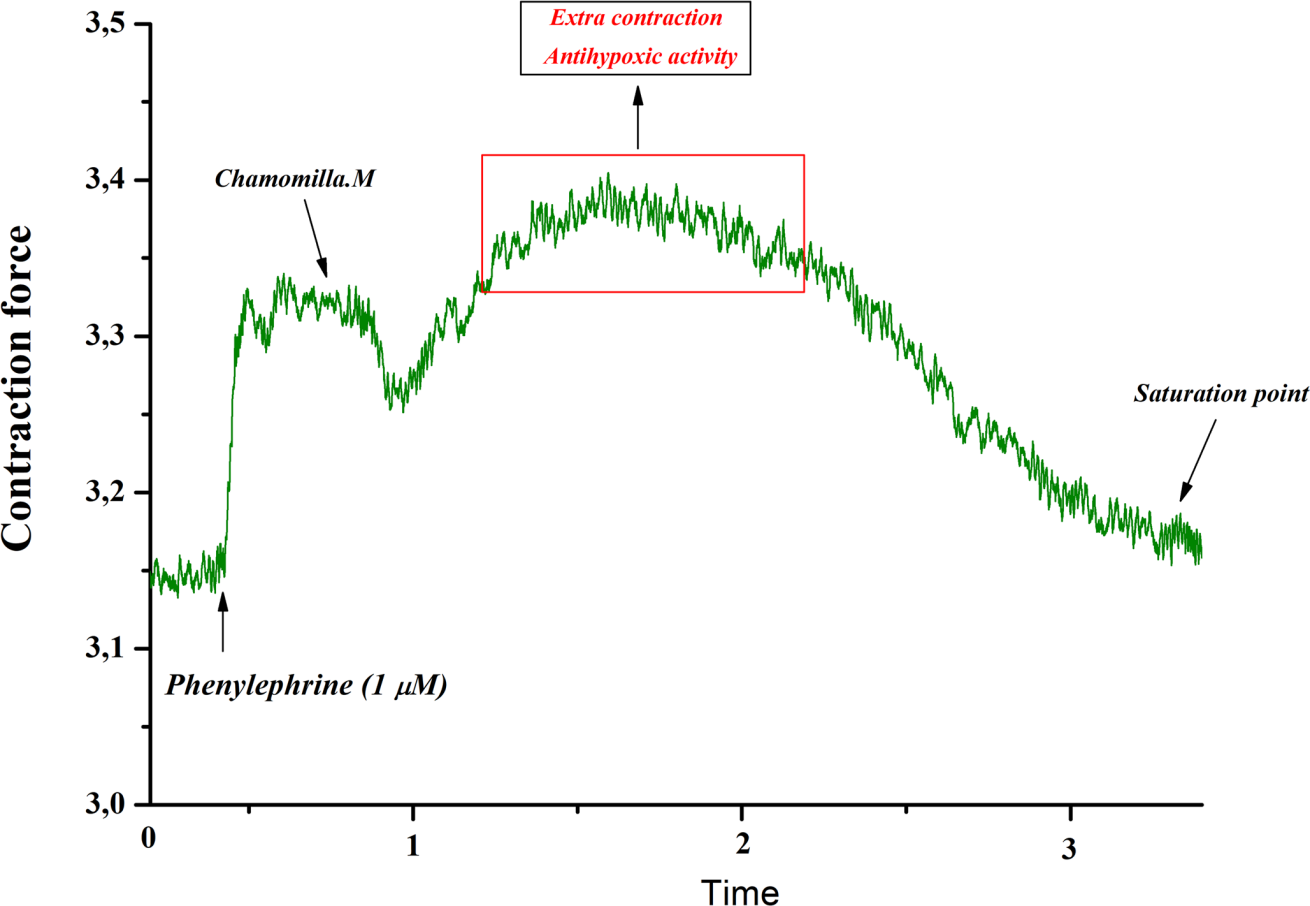
Original recording enhanced with originpro 8.5: Dose-dependent effect of *M. chamomilla* on contraction induced by phenylephrine

**Fig. 8:**
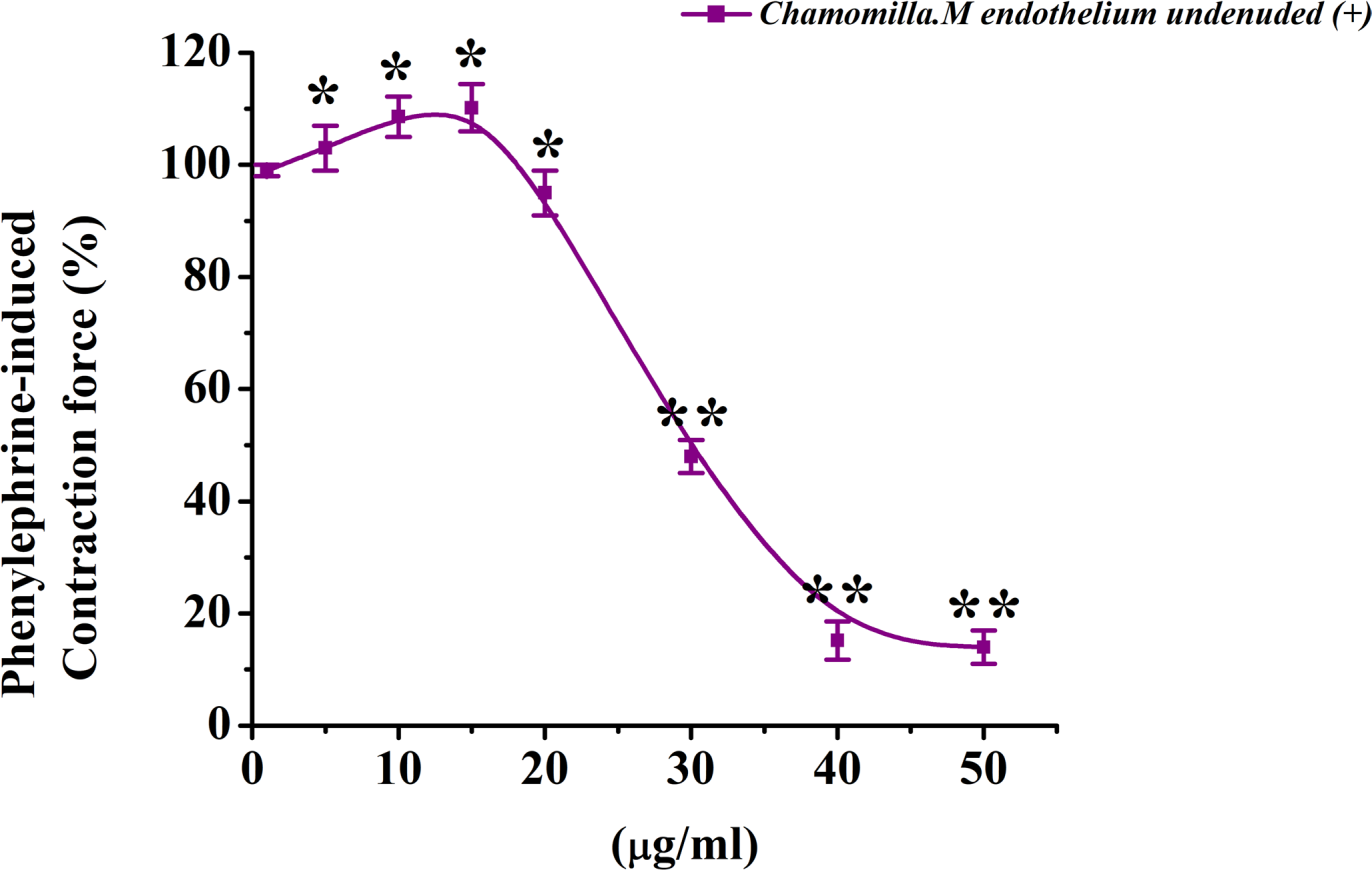
Dose-dependent effect of *M. chamomilla* extract on contraction of rat aorta induced by phenylephrine. The ordinate axis represents the contraction force of the aorta induced by 1 µM phenylephrine, taken as 100%. The abscissa axis represents the concentration of the extract. (Statistical significance in all cases: *p* < 0.05, *p* < 0.01; *n* = 5–7)

*M. chamomilla* extract, at its maximum concentration (50 µg/mL), significantly inhibited phenylephrine-induced contraction by 84.9 ± 3.8% compared to the control (Figs. 7 and 8).

**Fig. 9:**
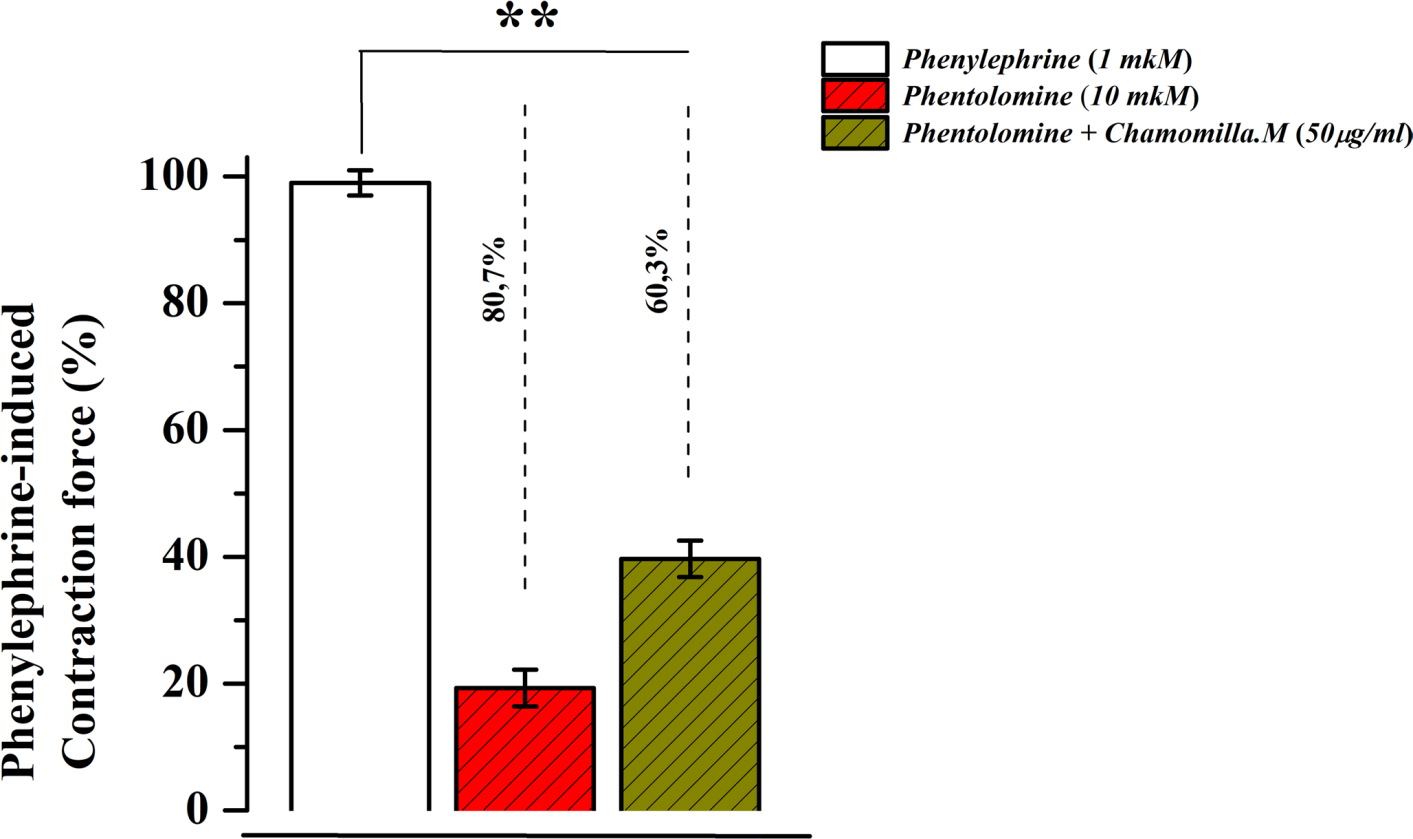
Effect of *M. chamomilla* extract on the relaxant effect of phentolamine (10 µM). The effect of *M. chamomilla* extract in the presence of 10 µM phentolamine. The contraction force of the aorta induced by 1 µM phenylephrine is taken as 100%. (Statistical significance: *p < 0.05, **p < 0.01; *n* = 6)

The effect of phentolamine (10 µM) on phenylephrine (1 µM)-induced contraction was examined, and it was found that phentolamine reduced the contraction force by 81.7 ± 3.1% compared to the control. Furthermore, when the effect of chamomile extract was tested in the presence of phentolamine, the contraction amplitude decreased to 39.1 ± 2.9% (Fig. 9). These findings suggest that the relaxant effect of *M. chamomilla *extract may be partially mediated through receptor mechanisms, and its interaction with phentolamine further clarifies this relationship.

**Fig. 10:**
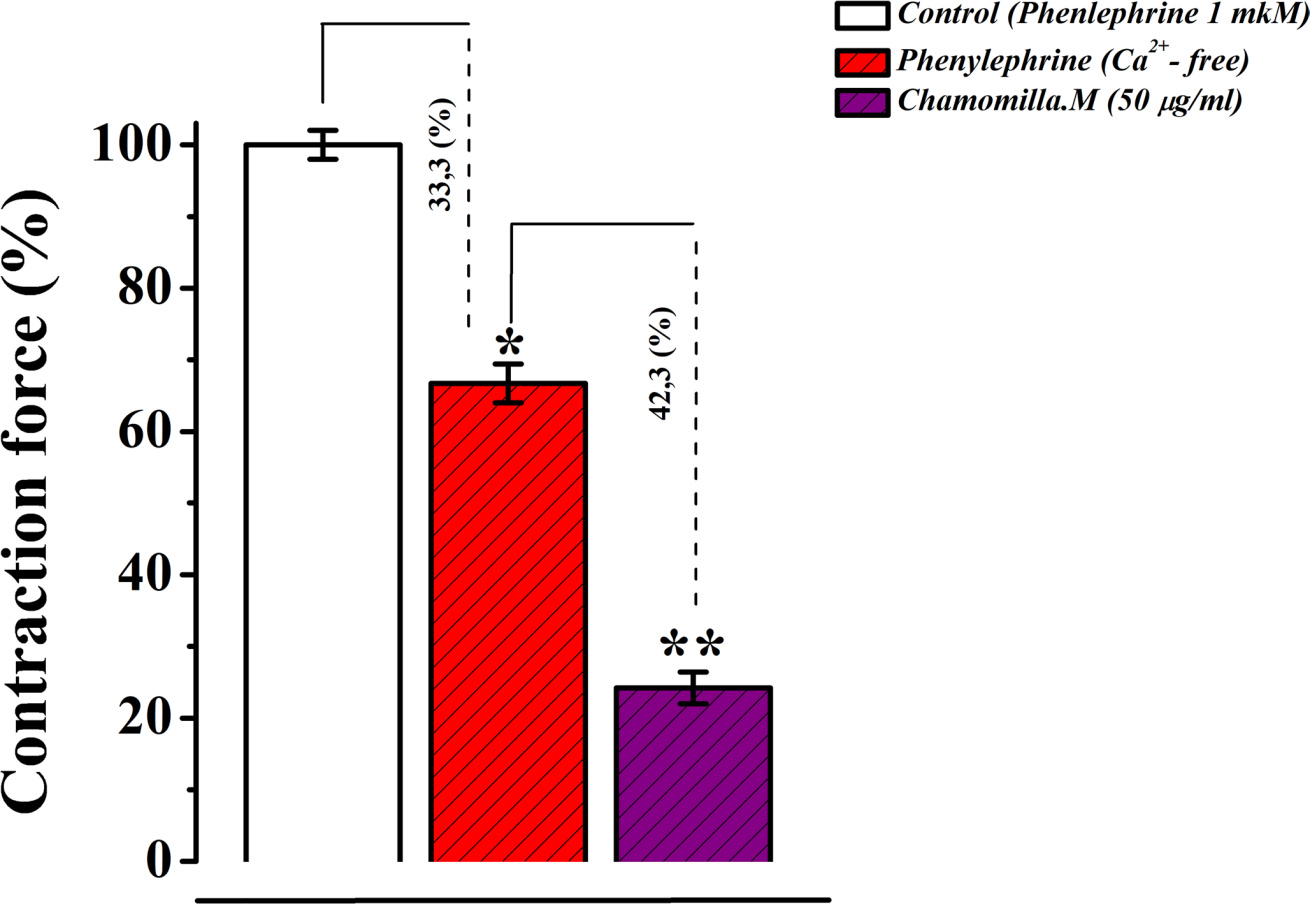
Relaxant effect of *M. chamomilla* extract on phenylephrine-induced contraction of Rat Aorta in Ca^2+-^free krebs solution. The contraction of the aorta induced by 1 µM phenylephrine in Ca2+-free Krebs solution is taken as 100% for control. (Statistical significance: *p < 0.05, **p < 0.01; *n* = 4–6)

The contraction induced by phenylephrine (1 µM) was determined to be 69 ± 3.1% of the contraction observed in normal Krebs solution, which was designated as 100% contraction. Under these experimental conditions, chamomile extract (50 µg/mL) significantly decreased the contraction amplitude by 30.5 ± 2.9% relative to the control (Fig. 10). These results indicate that chamomile extract may modulate Ca²⁺ release from the sarcoplasmic reticulum via the IP3 receptor (IP3R) pathway.

**Fig. 11:**
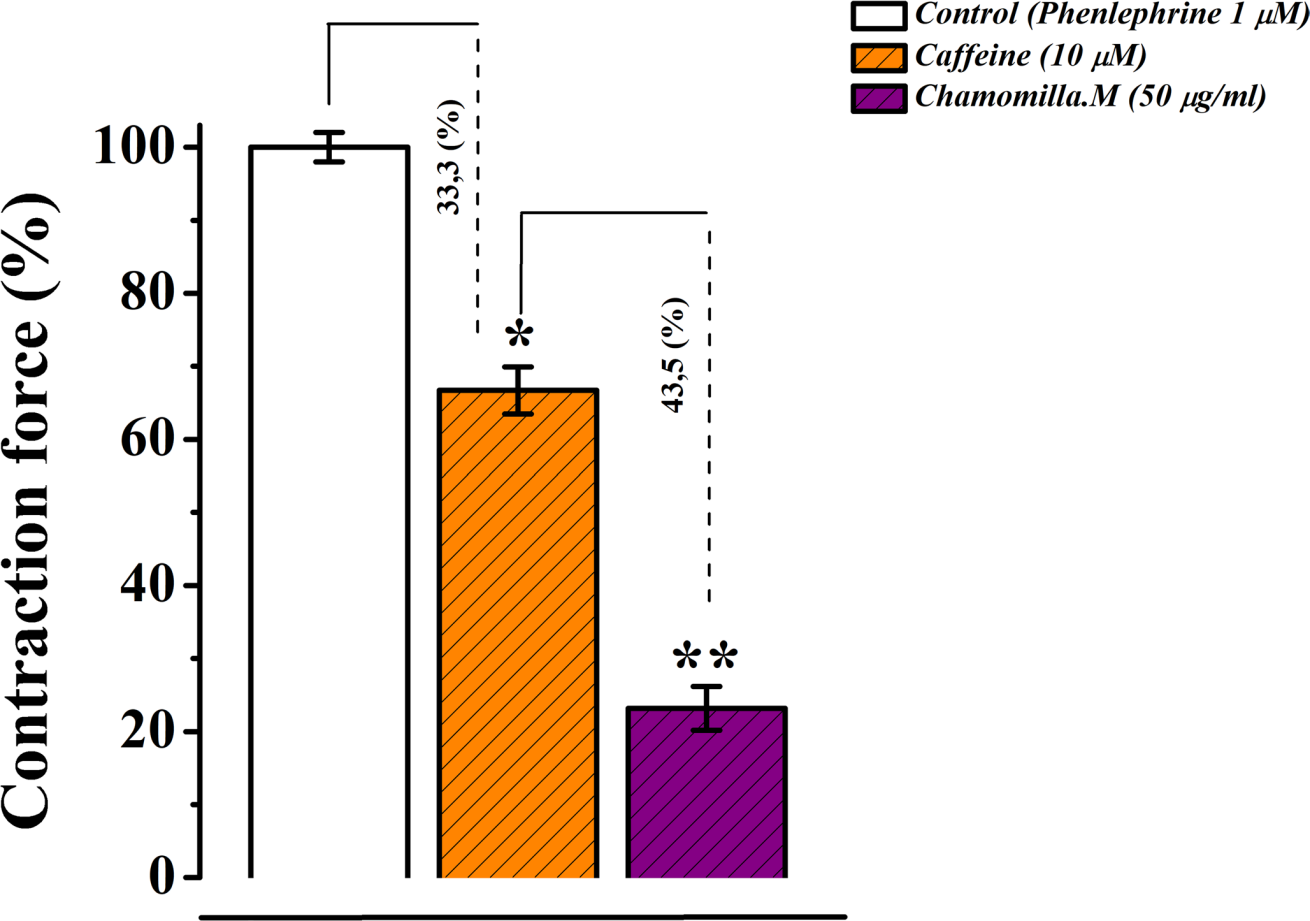
Relaxant effect of *M. chamomilla* extract on caffeine-induced contraction of rat aorta in normal krebs solution. The aorta contraction induced by 1 µM phenylephrine is taken as 100% for control. (Statistical significance: *p < 0.05, **p < 0.01; *n* = 6)

The contraction force induced by caffeine was used as an indicator of the Ca²⁺ ion content in the sarcoplasmic reticulum (SR). In normal Krebs solution containing 2.5 mM Ca²⁺, caffeine (10 mM) induced a contraction force corresponding to 62.5 ± 2.2% of the contraction induced by phenylephrine (1 µM). Under these conditions, *M.chamomilla* extract significantly reduced the caffeine-induced contraction by 15.2 ± 2.8% compared to the control (Fig. 11).

#### Endothelium-dependent effects of M. chamomilla extract: comparison with L-NAME blocker

**Fig. 12:**
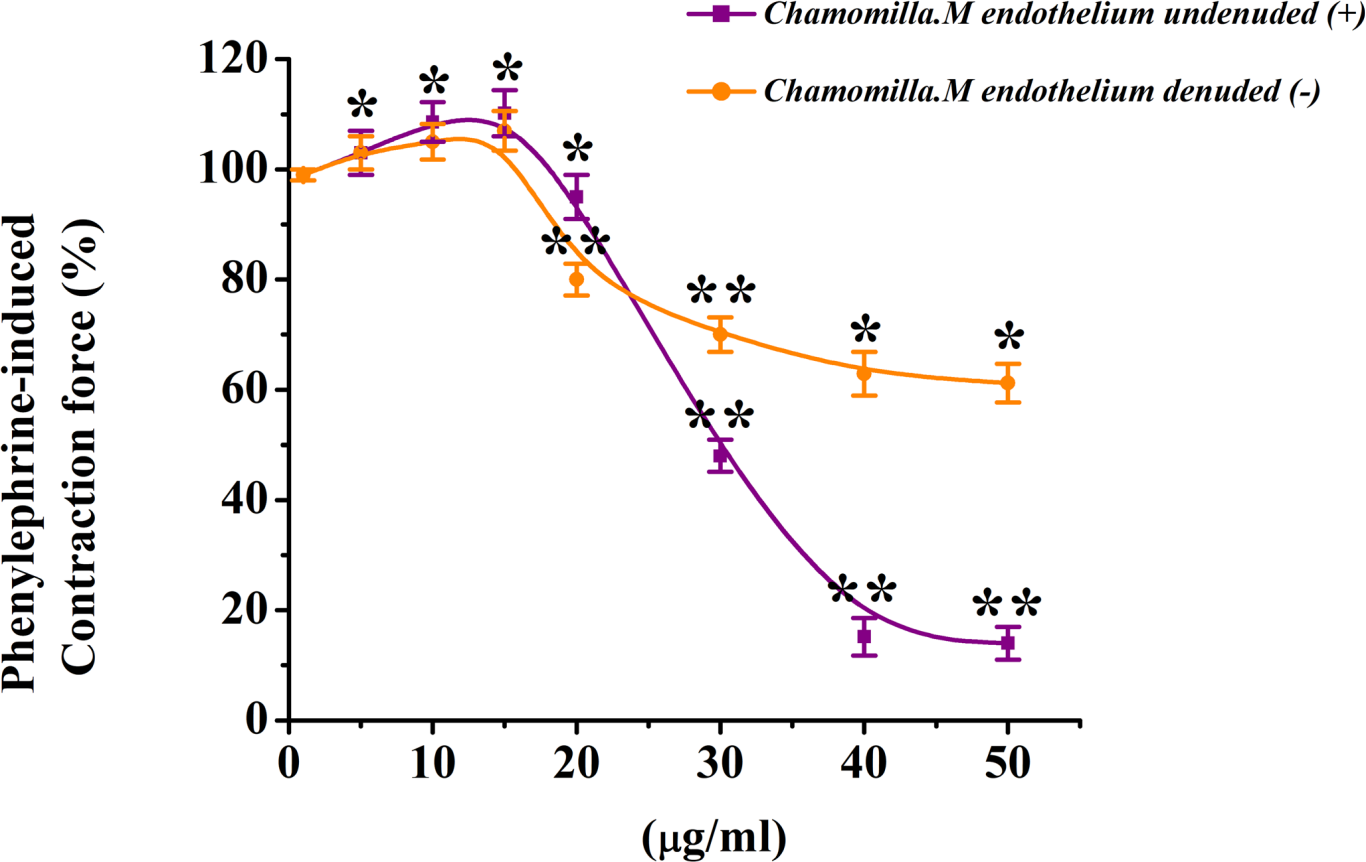
Concentration-dependent relaxant effect of *M. chamomilla* extract on phenylephrine-induced contraction in rat aorta with endothelium (+) and without endothelium (–). The contraction force induced by 1 µM phenylephrine is taken as 100% for control. (Statistical significance: *p < 0.05, **p < 0.01; *n* = 6)

In these conditions, a cocentration of chamomile extract (50 µg/mL) reduced contraction by 55.5 ± 3%, whereas in preparations with an intact endothelial layer, the reduction was only 20.6 ± 2.7% compared to the control (Fig. 12).

**Fig. 13:**
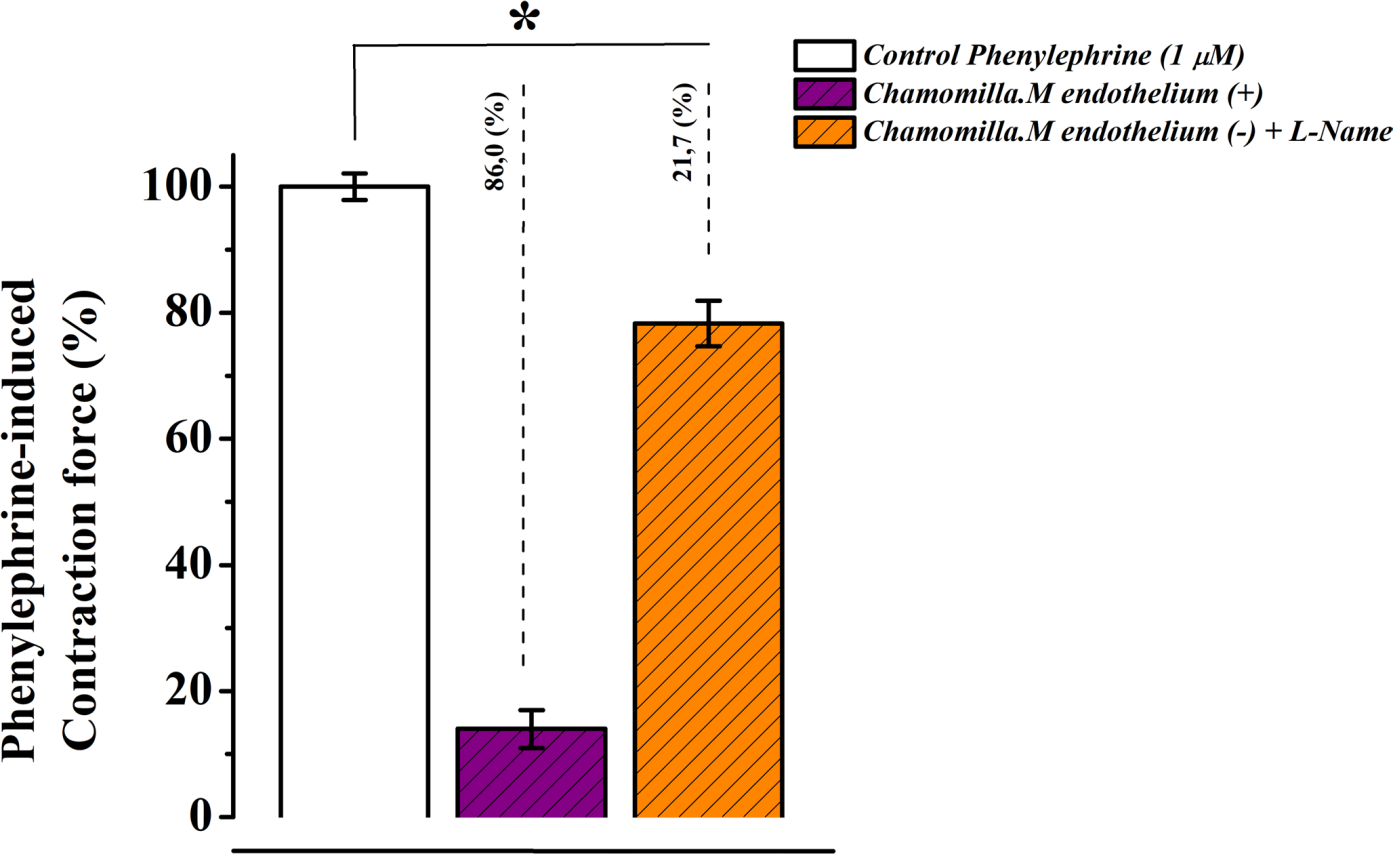
Concentration-dependent relaxant effect of *M. chamomilla* extract on rat aorta contraction in the presence of eNOS inhibitor L-NAME (100 µM). The contraction force induced by 1 µM phenylephrine is taken as 100% for control. (Statistical significance: *p < 0.05, **p < 0.01; *n* = 6)

In the presence of 100 µM L-NAME, the chamomile extract reduced the contraction force induced by phenylephrine (PE) by 55.5 ± 3%. In comparison, the reduction in contraction observed in preparations with an intact endothelial layer was only 13 ± 4%. (Fig. 13).

### Antihypertensive activity of *M. chamomilla* extract using the ‘’Tail Cuff’’ method: Determining the optimal dose and inducing hypertension with adrenaline hydrochloride

Initially, we determined the optimal dosage of *M. chamomilla* extract for our study. Rats were divided into four groups: the first group received 40 mg/kg, the second group 70 mg/kg, and the third group 100 mg/kg of the extract, administered orally. The fourth group served as a control (Table 1).

**Table 1:** Antihypertensive activity of *M. chamomilla* extract in vivo (M ± m = n3)

Arterial Blood Pressure (mmHg)						
Doses mg/kg	Control		An hour		Two hours	
	**SBP** (mmHg)	**DBP** (mmHg)	**SBP** (mmHg)	**DBP** (mmHg)	**SBP** (mmHg)	**DBP** (mmHg)
40	114 ± 15,0	77 ± 17,3	127,5 ± 10,2	91 ± 13,0	97,8 ± 17,8	70,5 ± 15,0
70	119,0 ± 18,2	85,0 ± 17,5	105 ± 20,6	83 ± 15,8	110 ± 18,5	87,8 ± 80,7
100	144 ± 15,3	110 ± 15,5	105 ± 15,4	74 ± 11,6	122 ± 13,6	90 ± 14,7

**Fig. 14:**
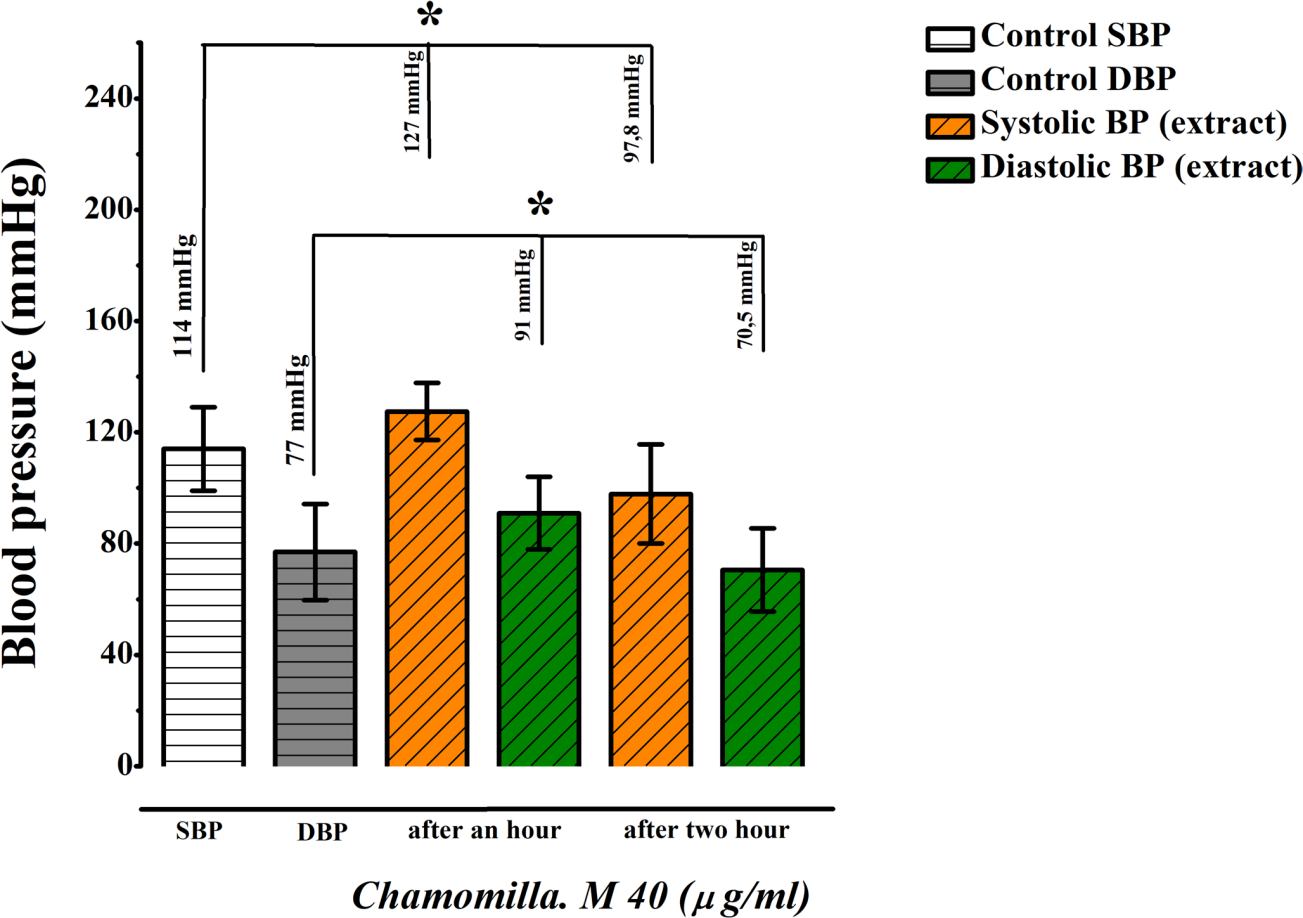
Dose-response activity of *M. chamomilla* extract at 40 mg/kg (Administered Orally, *n* = 3)

Following the administration, blood pressure measurements were recorded. At a dose of 40 mg/kg, the baseline systolic and diastolic pressures were 114 mmHg and 77 mmHg, respectively. One hour after administration, the systolic pressure rose to 127 mmHg, and the diastolic pressure increased to 91 mmHg, indicating the initial manifestation of the extract’s antihypoxic properties. By the second hour, the extract exerted a more pronounced effect, reducing systolic pressure to 97.8 mmHg and diastolic pressure to 70.5 mmHg (Fig. 14).

**Fig. 15:**
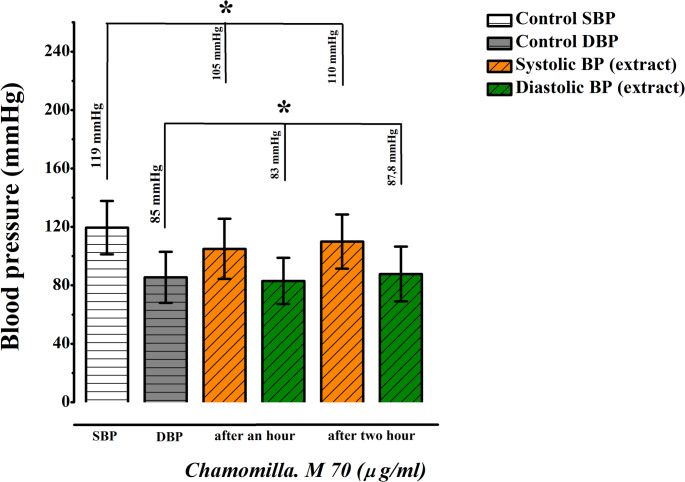
Dose-response activity of *M. chamomilla* extract at 70 mg/kg (Administered Orally, *n* = 3)

**Fig. 16:**
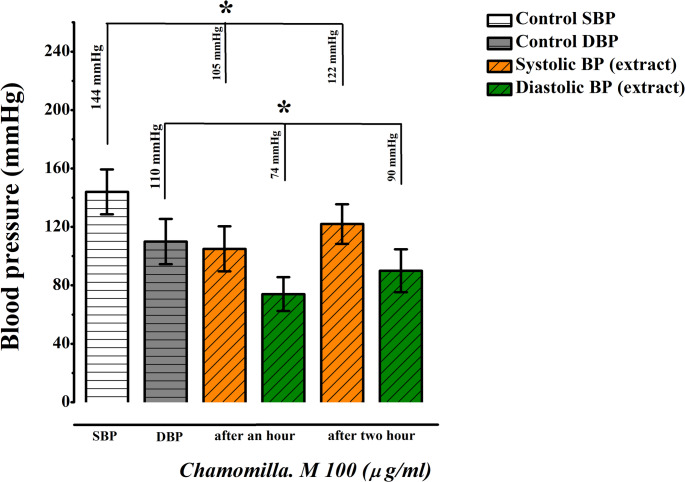
Dose-response activity of *M. chamomilla* extract at 100 mg/kg (Administered Orally, *n* = 3)

In contrast, the 70 mg/kg dose produced an unexpectedly rapid effect, resulting in agitation among the rats. Similarly, the 100 mg/kg dose caused adverse effects, including restlessness. Consequently, 40 mg/kg was identified as the optimal dose for further experiments due to its consistent and well-tolerated effects (Figs. 15 and 16).

In the second phase of the experiments, rats were again divided into three groups. The first group served as the control, the second group was treated with adrenaline hydrochloride to induce hypertension, and the third group was treated with adrenaline hydrochloride followed by 40 mg/kg of the extract. At least three rats were used in each group 

**Fig. 17:**
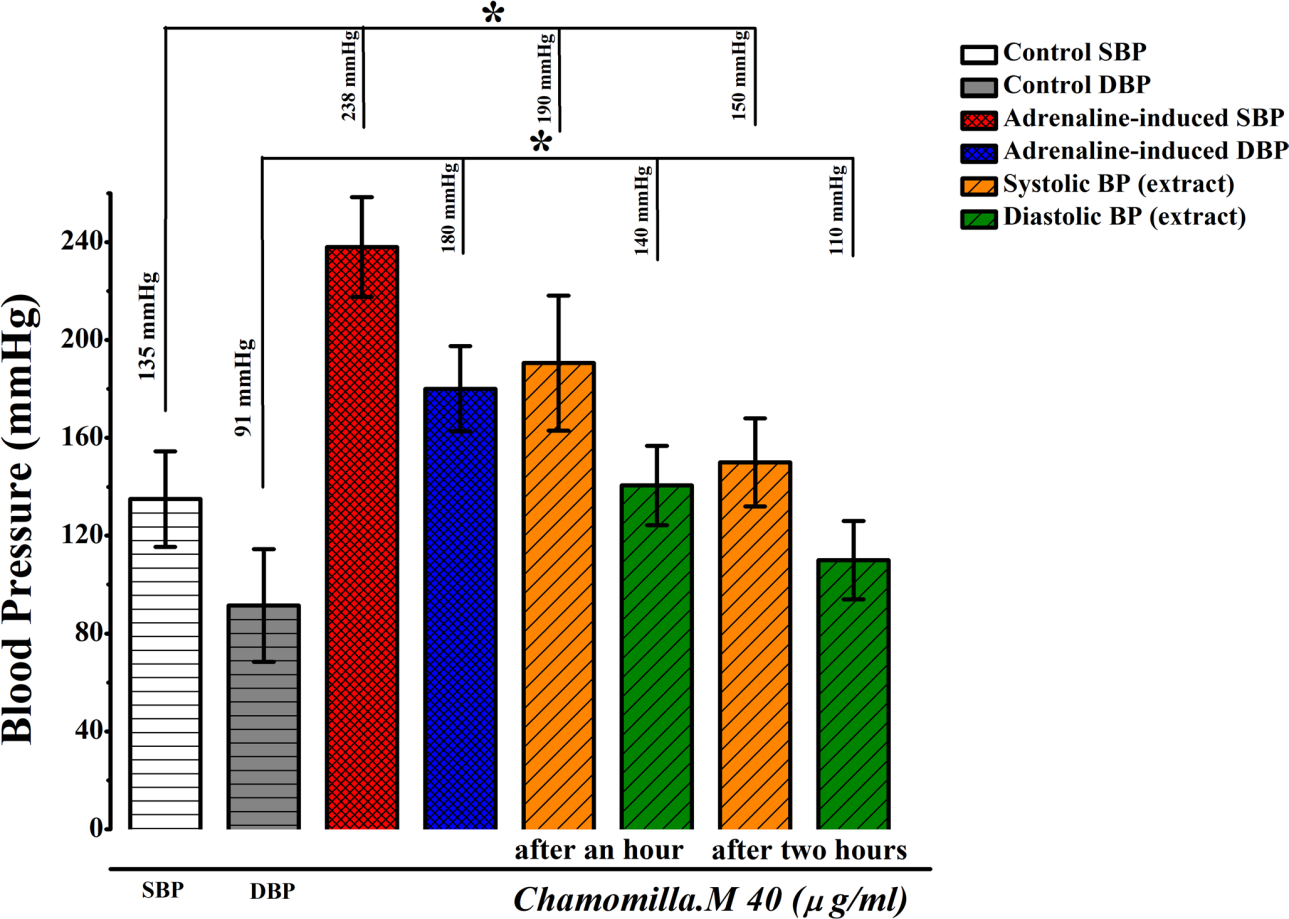
Hypertension induced with adrenaline hydrochloride via tail artery: evaluation of *M. chamomilla* extract activity at a 40 mg/kg dose over time (*n* = 3)

In the control group, the baseline systolic and diastolic pressures were 135 mmHg and 91 mmHg, respectively. Administration of adrenaline hydrochloride significantly increased systolic pressure to 238 mmHg and diastolic pressure to 180 mmHg. However, in hypertensive rats treated with the 40 mg/kg dose of the extract, systolic pressure dropped to 140 mmHg and diastolic pressure to 140 mmHg within the first hour. By the second hour, systolic pressure stabilized at 150 mmHg, while diastolic pressure decreased further to 110 mmHg (Fig. 17).

#### LC-MS based phytochemical constituents

Comprehensive phytochemical profiling of the plant extract was performed using liquid chromatography–mass spectrometry (LC-MS), which enabled the identification of a broad range of volatile, semi-volatile, and non-volatile constituents based on their retention times, molecular ions, and fragmentation patterns. Among the identified compounds were pinocarveol (C₁₀H₁₆O, 152.23 g/mol; RT 1.69 min), isobutyl acetate (C₆H₁₂O₂, 116.16 g/mol; RT 2.10 min), coumarin (C₉H₆O₂, 146.14 g/mol; RT 2.64 min), and p-cymene (C₁₀H₁₄, 134.22 g/mol; RT 2.99 min).

**Fig. 18:**
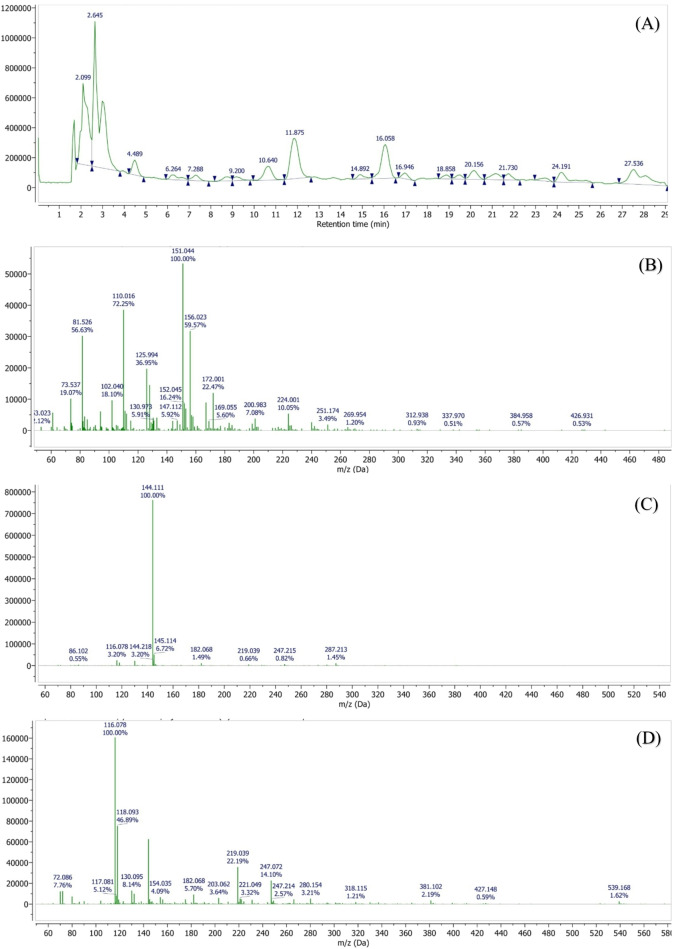

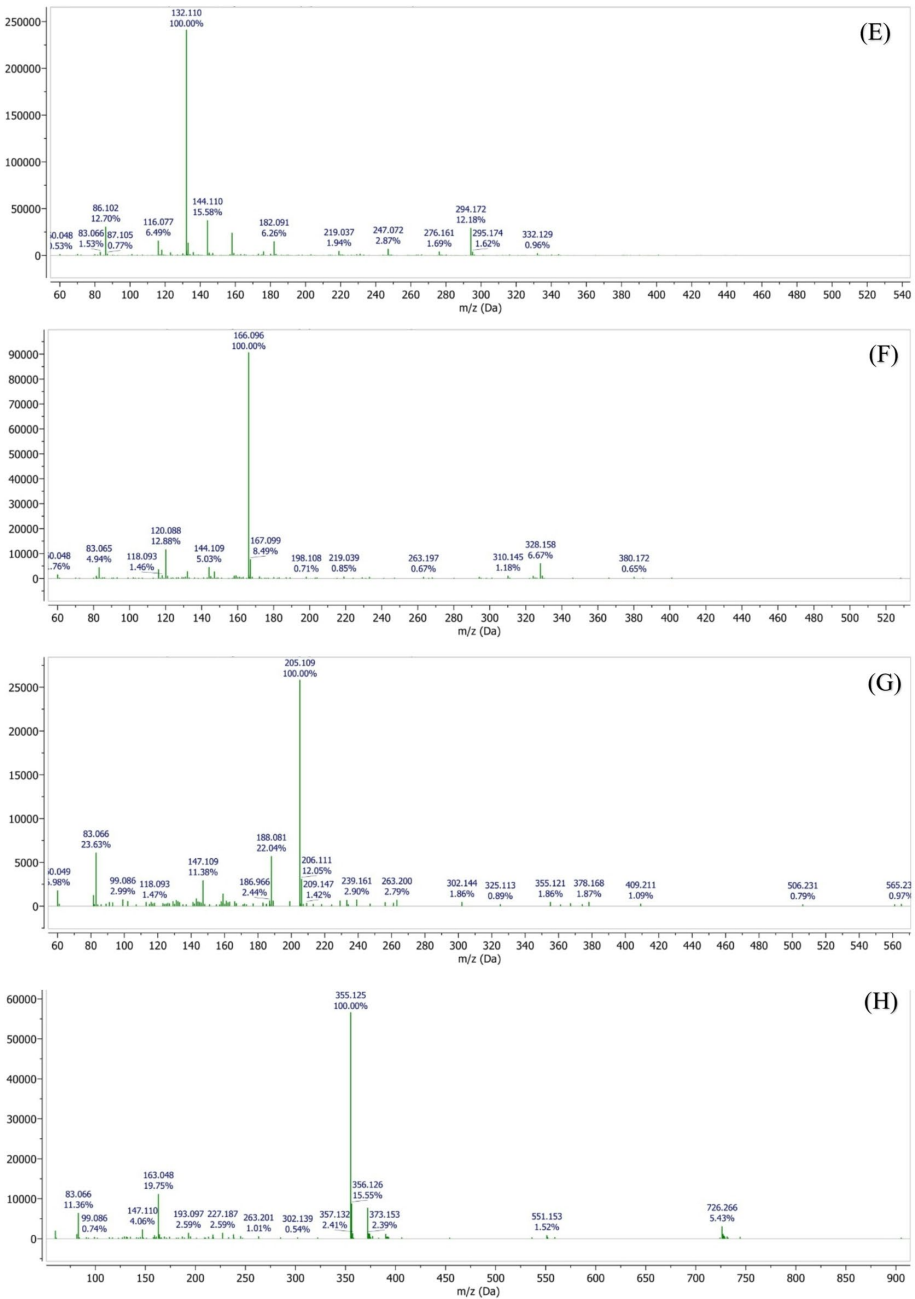

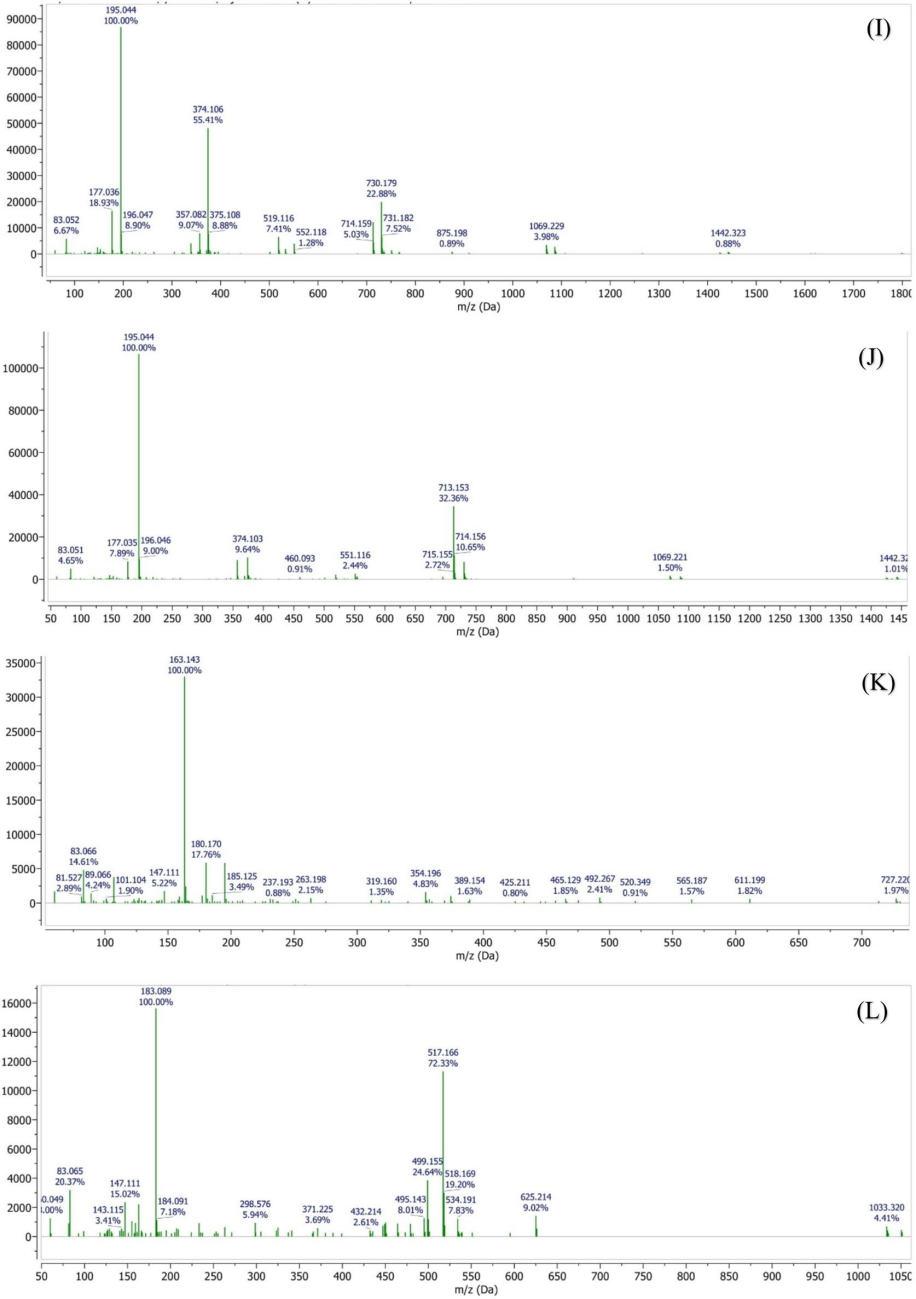

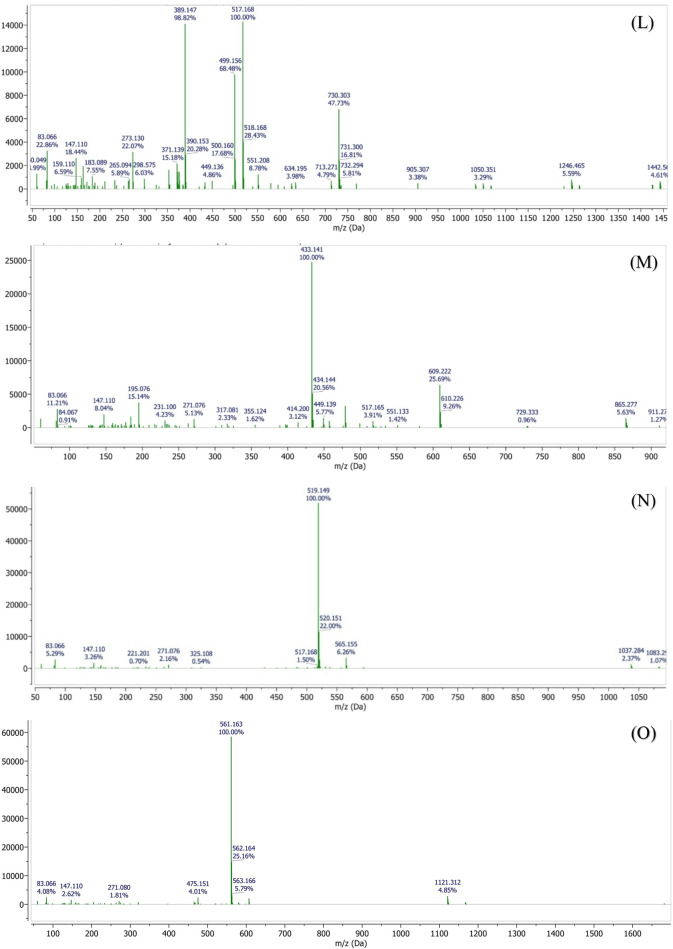
LC–MS chromatographic profiling of the plant extract. **(A)** Total ion chromatogram (TIC) obtained by LC–MS analysis. Peaks corresponding to detected phytochemical constituents are observed at retention times of **(B)** 1.69 min, **(C)** 2.64 min, **(D)** 2.09 min, **(E)** 2.99 min, **(F)** 4.49 min, **(G)** 7.36 min, **(H)** 10.6 min, **(I)** 11.8 min, **(J)** 16.0 min, **(K)** 16.9 min, **(L)** 19.4 min, **(M)** 20.1 min, **(N)** 21.1 min, and **(O)** 24.1 min. Individual peaks were assigned based on their retention times and corresponding mass spectral characteristics

The LC-MS analysis further revealed the presence of several phenolic acids, including vanillic acid (C₈H₈O₄, 168.15 g/mol), chlorogenic acid (C₁₆H₁₈O₉, 354.31 g/mol), ferulic acid (C₁₀H₁₀O₄, 194.18 g/mol), and p-coumaric acid (C₉H₈O₃, 164.16 g/mol). In addition, sesquiterpenes such as bisabolene (C₁₅H₂₄, 204.35 g/mol) and chamazulene (C₁₄H₁₆, 184.28 g/mol) were detected (Figure. 18).

Notably, high-molecular-weight polyphenolic compounds, including 4,5-O-dicaffeoylquinic acid and 1,5-dicaffeoylquinic acid (C₂₅H₂₄O₁₂, 516.45 g/mol), as well as flavonoid glycosides such as apigenin-7-O-glucoside (C₂₁H₂₀O₁₀, 432.38 g/mol) and apigenin-7-O-neohesperidoside (C₂₇H₃₀O₁₄, 578.52 g/mol), were successfully identified.

The identification of these chemically diverse constituents using LC-MS provides a reliable basis for subsequent evaluation of the extract’s pharmacological and biological activities (Table 2).

**Table 2:** Retention times and molecular characteristics of detected compounds

	Retention time		Name	Molecular Formula	Relative Molecular Mass
1	1.69		Pinocarveol	C_10_H_16_O	152.23 g/mol
2	2.099		Isobutyl acetate	C_6_H_12_O_2_	116.16 g/mol
3	2.64		Coumarin	C_9_H_6_O_2_	146.14 g/mol
4	2.99		p-Cymene	C_10_H_14_	134.22 g/mol
5	4.49		Vannilic acid	C_8_H_8_O_4_	168.15 g/mol
6	7.36	Bisabolene		C_15_H_24_	204.35 g/mol
7	10.64		Chlorogenic acid	C_16_H_18_O_9_	354.31 g/mol
8	11.87		Ferulic acid	C_10_H_10_O_4_	194.18 g/mol
9	16.9		p-Coumaric acid	C_9_H_8_O_3_	164.16 g/mol
10	19.47		Chamazulene	C_14_H_16_	184.28 g/mol
11	20.13		4,5-O-Dicaffeoylquinic acid	C_25_H_24_O_12_	516.45 g/mol
12	20.18		Apigenin-7-O-glucoside	C_21_H_20_O_10_	432.38 g/mol
13	24.15		1,5-Dicaffeoylquinic acid	C_25_H_24_O_12_	516.45 g/mol
14	27.5		Apigenin-7-O-neohesperidoside	C_27_H_30_O_14_	578.52 g/mol

#### Binding affinity and inhibitory potential of natural compounds via molecular Docking

**Fig. 19:**
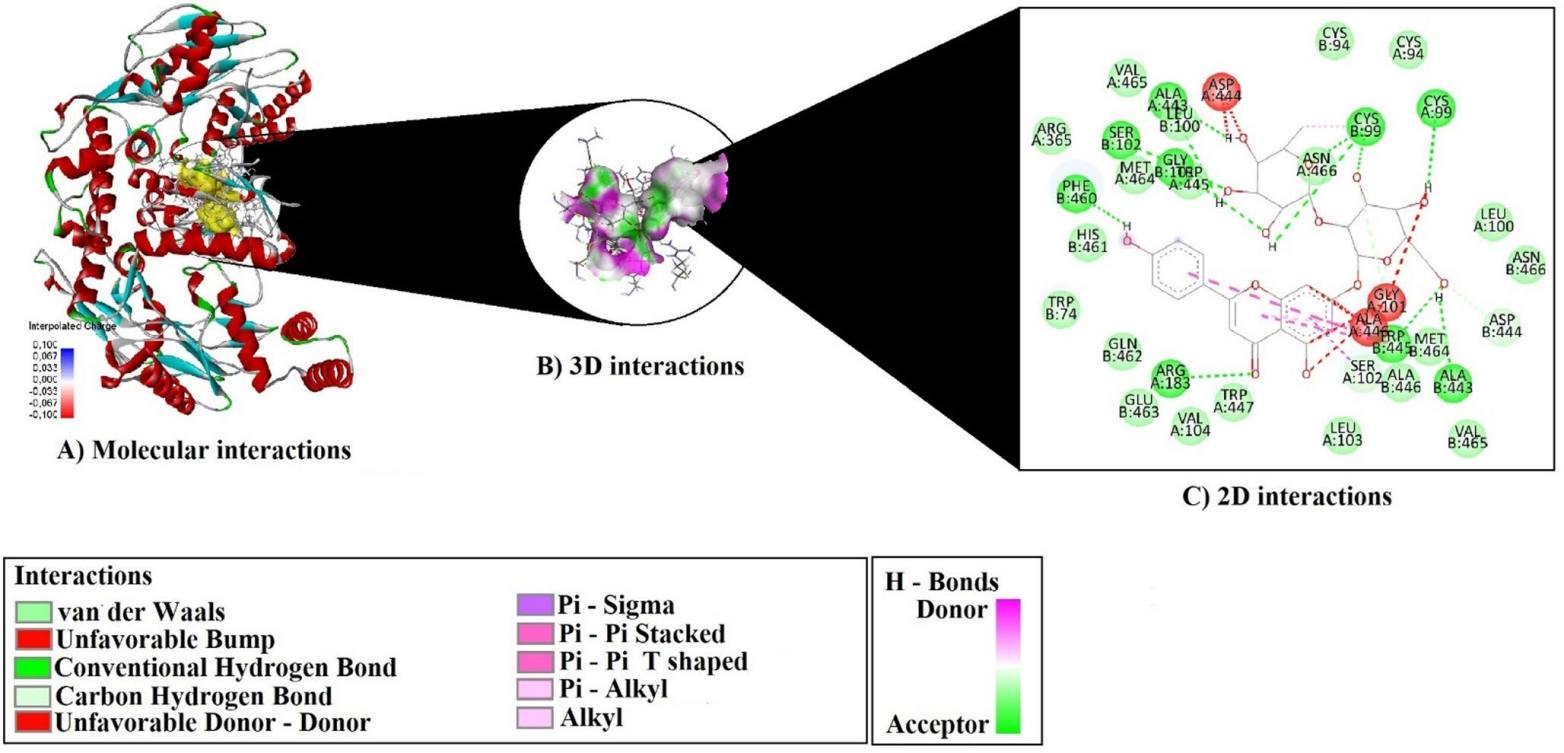
Molecular docking interactions of Apigenin-7-O-neohesperidoside with 7VF

**Fig. 20:**
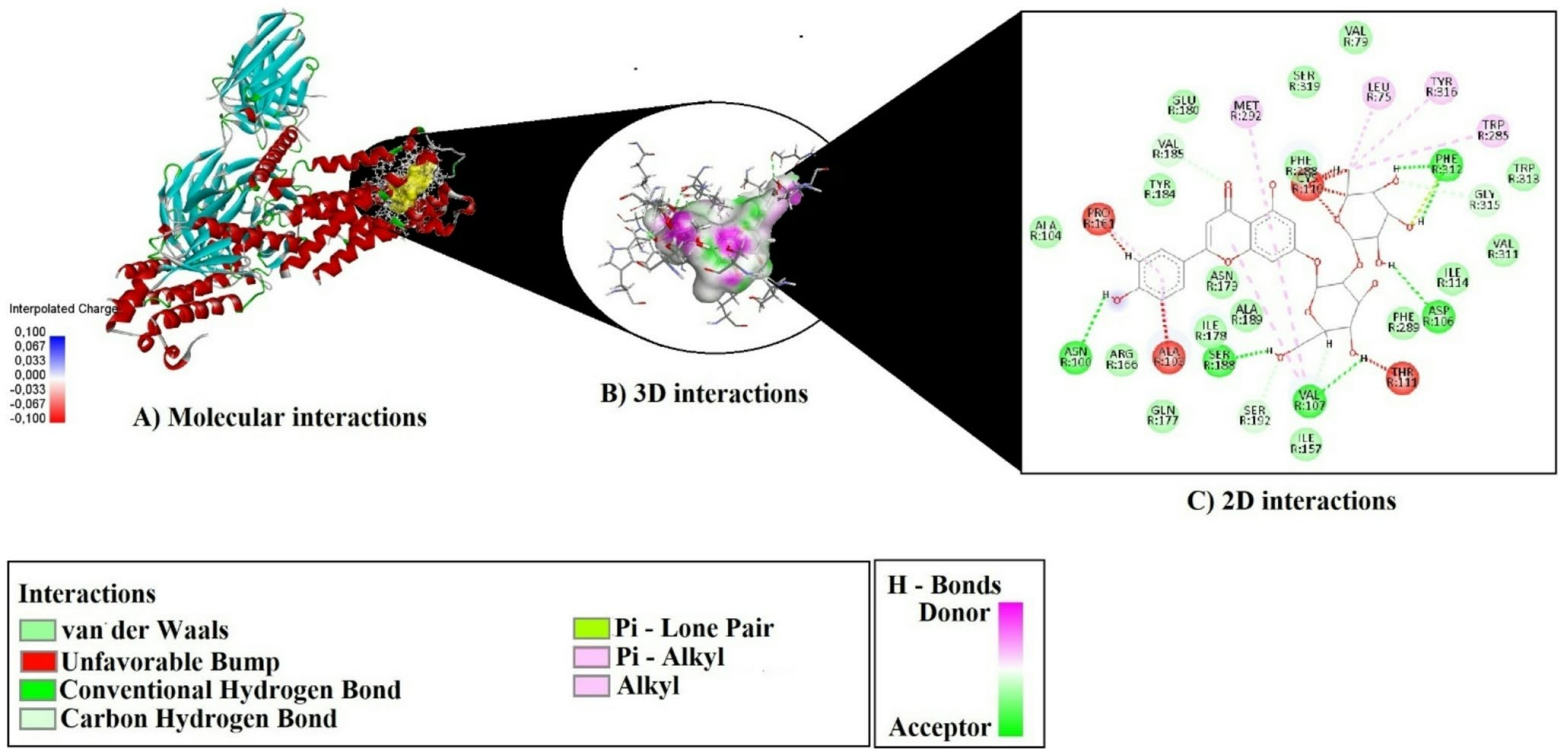
Molecular docking interactions of Apigenin-7-O-neohesperidoside with 8THK

**Fig. 21:**
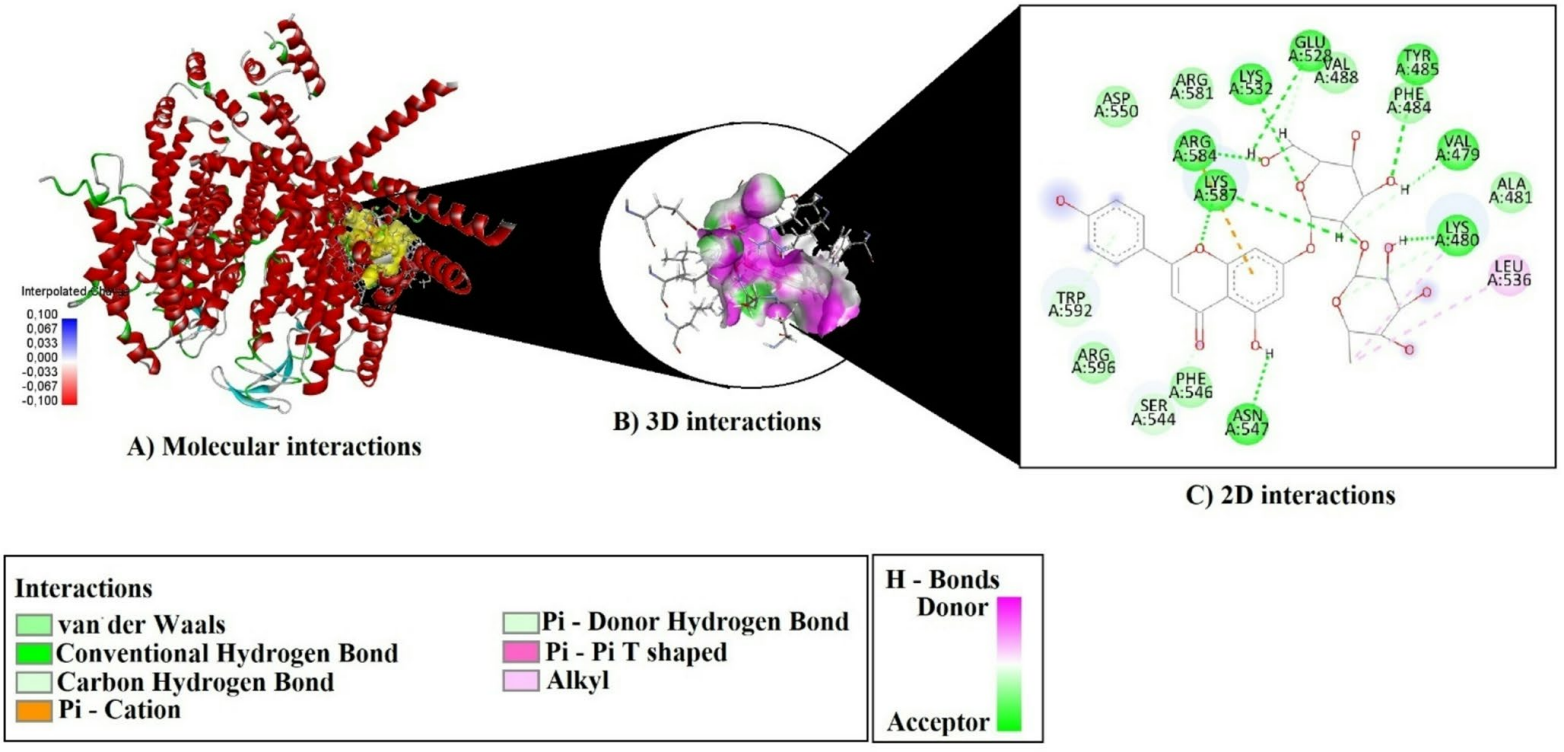
Molecular docking interactions of Apigenin-7-O-neohesperidoside with 3NOS

As a result of molecular docking analyses, the binding potentials of each phytochemical compound identified from the plant extracts were evaluated against three selected target proteins (7VFS, 8THK, and 3NOS) (Figs. 19 and 20–21). The assessment was based on key parameters such as binding energy (kcal/mol), ligand efficiency (LE), fit quality (FQ), binding efficiency index (BEI), and estimated inhibition constant (Ki, µM) (Table 3).

**Table 3:** Molecular Docking outcomes: binding energies, LE, FQ, BEI, and Ki values of phytochemicals

	Protein	Binding Energy (kcal/mol)	LE	FQ	BEI	Ki (µM)
Pinocarveol	7VFS	−5.5	0.500	0.403	0.036	92.475
	8THK	−6.2	0.563	0.454	0.040	28.353
	3NOS	−6.1	0.554	0.447	0.040	33.570
Isobutyl acetate	7VFS	−4.4	0.550	0.259	0.037	592.683
	8THK	−5.1	0.637	0.301	0.043	181.722
	3NOS	−4.9	0.612	0.289	0.042	254.740
Coumarin	7VFS	−6.6	0.600	0.484	0.045	14.428
	8THK	−6.9	0.627	0.506	0.047	8.693
	3NOS	−7.1	0.645	0.520	0.048	6.201
p-Cymene	7VFS	−7.0	0.700	0.485	0.052	7.342
	8THK	−6.9	0.690	0.478	0.051	8.693
	3NOS	−6.5	0.650	0.450	0.048	17.083
Vanillic Acid	7VFS	−5.6	0.466	0.429	0.333	78.105
	8THK	−6.1	0.508	0.468	0.036	33.570
	3NOS	−6.5	0.541	0.498	0.038	17.083
Bisabolene	7VFS	−7.6	0.506	0.637	0.036	2.665
	8THK	−8.1	0.540	0.679	0.038	1.145
	3NOS	−7.5	0.500	0.629	0.035	3.155
Chlorogenic acid	7VFS	−8.1	0.324	0.741	0.022	1.145
	8THK	−7.4	0.296	0.677	0.020	3.736
	3NOS	−9.0	0.360	0.824	0.025	0.250
Ferulic acid	7VFS	−6.3	0.450	0.516	0.032	23.947
	8THK	−6.7	0.478	0.549	0.034	12.186
	3NOS	−7.3	0.521	0.598	0.037	4.424
p-Coumaric acid	7VFS	−6.6	0.550	0.506	0.040	14.428
	8THK	−6.5	0.541	0.498	0.039	17.083
	3NOS	−7.0	0.583	0.537	0.042	7.342
Chamazulene	7VFS	−8.7	0.621	0.712	0.047	0.415
	8THK	−8.1	0.578	0.663	0.043	1.145
	3NOS	−8.3	0.592	0.680	0.045	0.817
4,5-O-Dicaffeoylquinic acid	7VFS	−8.8	0.237	0.790	0.017	0.351
	8THK	−7.6	0.205	0.682	0.014	2.665
	3NOS	−9.7	0.262	0.871	0.018	0.076
Apigenin-7-O-glucoside	7VFS	−9.4	0.303	0.858	0.021	0.127
	8THK	−8.1	0.261	0.739	0.018	1.145
	3NOS	−10.1	0.325	0.922	0.023	0.039
1,5-Dicaffeoylquinic acid	7VFS	−9.0	0.243	0.808	0.017	0.250
	8THK	−7.5	0.202	0.673	0.014	3.155
	3NOS	−9.1	0.245	0.817	0.017	0.211
Apigenin-7-O-neohesperidoside	7VFS	−10.3	0.251	0.911	0.017	0.027
	8THK	−8.3	0.202	0.734	0.014	0.817
	3NOS	−10.6	0.258	0.938	0.018	0.016

* BEI: Binding Efficiency Index, FQ: Fit Quality, Ki: Estimated Inhibition Constant, LE: Ligand Efficiency

The monoterpene pinocarveol exhibited its strongest interaction with the 8THK protein, with a binding energy of − 6.2 kcal/mol and a corresponding Ki value of 28.353 µM. In contrast, isobutyl acetate showed relatively weak binding affinity, especially toward 8THK (–5.1 kcal/mol; Ki: 181.722 µM), indicating limited inhibitory potential.

The aromatic lactone coumarin showed a favorable binding profile, particularly with 3NOS (–7.1 kcal/mol; Ki: 6.201 µM), while p-cymene demonstrated strong affinity with 7VFS (–7.0 kcal/mol; Ki: 7.342 µM).

Among the phenolic acids, vanillic acid exhibited moderate binding to 3NOS (–6.5 kcal/mol), whereas ferulic acid demonstrated stronger interaction with the same protein (–7.3 kcal/mol; Ki: 4.424 µM).

Bisabolene displayed notable binding across all three targets, with its most potent interaction observed with 8THK (–8.1 kcal/mol; Ki: 1.145 µM). Chlorogenic acid also showed high inhibitory potential, especially against 3NOS (–9.0 kcal/mol; Ki: 0.250 µM).

High molecular weight compounds such as 4,5-O-dicaffeoylquinic acid and 1,5-dicaffeoylquinic acid exhibited strong binding energies with 3NOS (–9.7 and − 9.1 kcal/mol, respectively), resulting in low Ki values of 0.076 µM and 0.211 µM.

Among the flavonoid derivatives, apigenin-7-O-glucoside and apigenin-7-O-neohesperidoside emerged as the most potent inhibitors, particularly against 3NOS, with binding energies of − 10.1 and − 10.6 kcal/mol and remarkably low Ki values of 0.039 µM and 0.016 µM, respectively.

Overall, flavonoid glycosides and caffeoylquinic acid derivatives demonstrated high inhibitory potential due to their low binding energies and Ki values. Notably, apigenin-7-O-neohesperidoside exhibited the most promising results, with binding energies of − 10.3, − 8.3, and − 10.6 kcal/mol against 7VFS, 8THK, and 3NOS, respectively. Its exceptionally low Ki value (0.016 µM), combined with high FQ scores (0.911–0.938) and stable interaction profiles, suggests a strong biological affinity for the studied targets. Therefore, apigenin-7-O-neohesperidoside stands out as a leading candidate for further investigation due to its high therapeutic potential and strong binding affinities toward key protein targets.

Molecular docking analysis revealed that Apigenin-7-O-neohesperidoside formed extensive and diverse interactions with all three target proteins—7VFS, 8THK, and 3NOS—including conventional hydrogen bonds, π–π stacking, and hydrophobic contacts (Table 4).

**Table 4:** Hydrogen Bonding, π–π, and hydrophobic interactions observed in docked complexes of Apigenin-7-O-neohesperidoside

Compounds	Protein	H-Bond	Pi–Pi Stacking	Alkyl Interactions
Apigenin-7-O-neohesperidoside	7VFS	TYR485:HN–O2 LYS532:HZ1–O4 ARG584:HH21–O10 LYS587:HZ3–O6 LYS587:HZ2–O1 H1–VAL479:O H3–ASN547:OD1 H13–GLU528:OE2 H22–LYS480:O LYS480:HA–O11 LYS532:HE2–O4 SER544:HB1–O7 H14–GLU528:OE2 H19–VAL479:O H29–O5	TRP592 (2× T-shaped)	C27–LYS480 (Alkyl), C27–LEU536 (Alkyl)
	8THK	H2–VAL107:O H3–O7 H6–ASN100:O H13–SER188:OG H20–PHE312:O H21–PHE312:O H22–ASP106:OD2 VAL185:HA–O7 SER192:HB1–O10 SER192:HB2–O10 GLY315:HA2–O12 H16–VAL107:O H23–O5	-	C27–LEU75 (Alkyl) TRP285 (Pi–Alkyl) TYR316 (Pi–Alkyl) VAL107 (2× Pi–Alkyl) MET292 (Pi–Alkyl) ALA103 (Pi–Alkyl) PRO161 (Pi–Alkyl)
	3NOS		TRP445 (2×Pi–Pi Stacked + 4×Pi–Pi T-shaped)	C27–CYS99 (Alkyl) ALA446 (Pi–Alkyl)

With 7VFS, the compound established a total of 15 hydrogen bonds, involving residues such as Tyr485, Lys480, Lys532, Lys587, Arg584, Asn547, Glu528, and Ser544. These interactions occurred through both backbone and side-chain atoms. Additionally, two T-shaped π–π stacking interactions were observed with Trp592, and alkyl interactions were identified between the ligand’s C27 atom and residues Lys480 and Leu536, suggesting significant hydrophobic stabilization within the binding pocket.

In the case of 8THK, the ligand engaged in 13 hydrogen bonds, notably with Val107, Asn100, Ser188, Phe312, Asp106, Ser192, and Gly315. A range of hydrophobic π–alkyl contacts were also formed with residues such as Trp285, Tyr316, Val107, Met292, Ala103, and Pro161, alongside one alkyl interaction with Leu75 via the C27 position of the ligand.

For 3NOS, Apigenin-7-O-neohesperidoside demonstrated the most complex interaction profile, forming 16 hydrogen bonds with critical residues including Arg183, Cys99, Gly101, Ser102, Trp445, Phe460, Ala443, and Asp444. Particularly notable were the six π–π interactions with Trp445, comprising two stacked and four T-shaped geometries. Furthermore, the ligand engaged in alkyl interaction with Cys99 and π–alkyl interaction with Ala446.

These findings highlight the ligand’s high affinity and structural complementarity with all three proteins. The abundance and diversity of interactions support the compound’s strong binding stability and suggest significant biological potential across multiple targets.

## Discussion

The contractile activity of aortic preparations under the influence of 50 mM KCl is known to be primarily mediated by the activation of voltage-dependent Ca²⁺ channels located on the plasma membrane of smooth muscle cells. The increase in extracellular K⁺ concentration leads to changes in membrane potential, causing membrane depolarization and triggering the activation of voltage-gated Ca²⁺ channels. This results in an increased influx of Ca²⁺ ions into the cytoplasm, thereby enhancing contractile force(Abdullaev et al. 2024). In this context, the present study investigated the effects of *M. chamomilla* extract on rat aortic contractions induced by 50 mM KCl. The results revealed a concentration-dependent relaxant effect of the extract. At a concentration of 10 µg/mL, an increase in contractile activity was observed compared to the control group, suggesting a possible antihypoxic property of the extract (Zaripova et al. 2024).

The extract exerted a significant relaxant effect on KCl-induced contractions in rat aortic preparations. As previously stated, this effect may be mediated through the modulation of voltage-dependent Ca²⁺ channels (Fisslthaler et al. 2000). While KCl alone does not induce contraction in a Ca²⁺-free Krebs solution, varying the Ca²⁺ concentration (0–2.5 mM) leads to dose-dependent contractions (Khushmatov et al. 2020). In our experiments, when the Ca²⁺ concentration in Krebs solution containing 50 mM KCl was altered, dose-dependent contractions were recorded in the aortic tissues. Under these conditions, the *M. chamomilla* extract significantly reduced aortic contractility compared to the control, indicating an inhibitory effect on Ca²⁺ influx.

In conclusion, the extract effectively inhibited Ca²⁺ entry through voltage-dependent Ca²⁺ channels in the cell membrane, resulting in a relaxant effect on KCl-induced contractions. This inhibitory action reduces intracellular Ca²⁺ levels, thereby limiting contractile capacity and helping to alleviate spasms or excessive contractions in vascular smooth muscle.

To further clarify the involvement of voltage-dependent Ca²⁺ channels in the observed relaxant effects, additional experiments were conducted using verapamil, a specific L-type Ca²⁺ channel blocker (Izzatullo et al., 2024). In these experiments, a submaximal contractile concentration of verapamil (0.1 µM) was applied to KCl (50 mM)-induced aortic preparations. When the effects of verapamil (0.1 µM) and the extract (at its EC₅₀) were compared, the extract produced a more pronounced reduction in aortic contractile amplitude than the control group.

These findings suggest that verapamil may assist in blocking Ca²⁺ influx through voltage-dependent Ca²⁺ channels in smooth muscle cells, like the tested extract. Moreover, the data obtained from verapamil treatment provide valuable insight into the mechanism of action of the extract and allow for a deeper investigation of the role of voltage-dependent Ca²⁺ channels. Taken together, the relaxant effect observed is not solely limited to voltage-dependent Ca²⁺ channels; other ion transport mechanisms may also be involved. Further experiments are needed to elucidate these additional pathways in greater detail.

It is well established that various complex mechanisms are involved in the contraction of smooth muscle cells in blood vessels. These processes include not only voltage-dependent Ca²⁺ L-type channels but also the Ca²⁺ transport systems on the sarcoplasmic reticulum (SR), which play a crucial role in regulating intracellular calcium ion distribution and maintaining calcium homeostasis. These systems are critical in modulating the contractile capacity of the cell. The importance of these channels and Ca²⁺ transport systems in the function of vascular smooth muscle cells has been comprehensively examined in numerous scientific studies, and continued research in this area remains necessary(Eid et al. 2018).

For this reason, the relaxant effect on phenylephrine (1 µM)-induced contraction, an α-adrenergic agonist, was investigated. Phenylephrine (1 µM) increases intracellular Ca²⁺ levels by promoting Ca²⁺ release from the sarcoplasmic reticulum and facilitating Ca²⁺ influx through receptor-operated channels (Webb 2003). Consistent with the findings of Webb’s study, our results confirmed that phenylephrine (1 µM) increases intracellular Ca²⁺ levels by promoting Ca²⁺ influx, while *M. chamomilla* extract, at its maximum concentration (50 µg/mL), significantly reduced phenylephrine-induced contraction compared to the control group.

These findings suggest that the effects of chamomile extract may be mediated through receptor-operated mechanisms. To clarify this hypothesis, its interaction with phentolamine, a specific blocker of these channels, was assessed in subsequent experiments. In the absence of phentolamine, *M. chamomilla* extract (50 µg/mL) was again observed to reduce phenylephrine (1 µM)-induced contraction, consistent with previous findings(Zoirovich et al. 2024). When the effect of phentolamine (10 µM) on phenylephrine-induced contraction was examined, it was found that phentolamine reduced the contraction force compared to the control group. Moreover, when the effect of *M. chamomilla* extract was tested in the presence of phentolamine, the contraction amplitude decreased to 39.1 ± 2.9%. This indicates that the relaxant effect of chamomile extract may be partially mediated through receptor mechanisms, and the interaction with phentolamine further clarifies this relationship.

These findings suggest that the relaxant activity of the tested extract may be associated with the blockade of receptor-controlled Ca²⁺ channels. The experiments conducted using the α-adrenergic receptor blocker phentolamine support this conclusion.

In subsequent experiments, the effect of chamomile extract on the release of Ca²⁺ ions from the sarcoplasmic reticulum via the inositol 1,4,5-trisphosphate receptor (IP3R) was investigated. In these experiments, under conditions where Ca²⁺ ions were absent in the incubation medium, the phenylephrine (1 µM)-induced contraction was shown to be associated with Ca²⁺ release from the SR through the IP3R pathway (Fan et al. 2015). In our study, under phenylephrine (1 µM)-induced conditions, the administration of *M. chamomilla* extract (50 µg/mL) significantly reduced the contraction amplitude compared to the control group. These findings suggest that *M. chamomilla* e extract may influence the SR Ca²⁺ release process via the IP3R pathway.

The results indicate that the relaxant effect of *M. chamomilla* extract on the contractile activity of the aortic preparation in the absence of extracellular Ca²⁺ in the Krebs solution is likely associated with the inhibition of Ca²⁺ release from the SR through the IP3R pathway. According to the literature, caffeine triggers Ca²⁺ release from the SR in smooth muscle cells by activating ryanodine receptors (RyRs)(Li et al. 2008). In normal Krebs solution (2.5 mM Ca²⁺), caffeine (10 mM) produced a contraction force 62.5 ± 2.2% of that induced by phenylephrine (1 µM). Under these conditions, *M. chamomilla* extract significantly reduced the caffeine-induced contraction compared to the control group. These results suggest that *M. chamomilla* extract reduces contraction force, likely by modulating the release of Ca²⁺ ions from the SR via the IP3R and/or RyR pathways (Panklai et al. 2024).

Overall, the findings reveal that *M. chamomilla* extract exhibits a pronounced relaxant effect particularly under conditions of Ca²⁺ depletion. The extract reduces phenylephrine (1 µM)-induced contractile force by inhibiting Ca²⁺ release from the SR via the IP3R pathway. This observation provides further evidence that the action of *M. chamomilla* extract may involve modulation of IP3R function. Inhibition of IP3R reduces SR Ca²⁺ release, thereby lowering intracellular Ca²⁺ concentration ([Ca²⁺]i), which ultimately diminishes both relaxation and contraction responses in smooth muscle cells.

Endothelial dysfunction is characterized by an imbalance in mediators that regulate endothelial function and is often associated with vascular diseases (Li et al. 2018). While *M. chamomilla* extract (50 µg/mL) significantly reduced contraction, this decrease was only 20.6 ± 2.7% in preparations with intact endothelium compared to the control group. These findings suggest that the vasorelaxant effect of *M. chamomilla* extract may have therapeutic potential in conditions involving endothelial dysfunction, by acting on both vascular smooth muscle and the endothelium to help restore vascular function(Konstantinovsky et al. 2019).

*M. chamomilla* extract exhibited a pronounced effect in endothelium-denuded aortic rings, indicating that its vasorelaxant activity may be at least partially mediated by endothelium-dependent mechanisms(Kameni et al. 2019). Furthermore, incubation with L-NAME revealed that the vasorelaxant effect of the extract was significantly diminished. In the presence of 100 µM L-NAME, *M. chamomilla* extract reduced phenylephrine (PE)-induced contraction. In comparison, the reduction in contraction observed in preparations with intact endothelium was only 13 ± 4%. These results suggest that the relaxant effect of *M. chamomilla* extract is at least partly mediated via endothelial nitric oxide synthase (eNOS)-dependent mechanisms, likely involving the production of nitric oxide (NO).

The attenuation of the extract’s effect in the presence of L-NAME further supports the critical role of endothelial function particularly NO in its relaxant properties. This highlights the potential therapeutic applications of *M. chamomilla* extract in managing vascular dysfunction and endothelium-related conditions (Brüll et al. 2015).

Overall, these findings emphasize the role of NO production in the vasorelaxant effect of the extract and support the hypothesis that its mechanism of action is closely related to endothelial function and the NO–cGMP signaling pathway. This provides a significant foundation for the potential therapeutic use of the extract in managing conditions associated with vascular smooth muscle contraction and endothelial dysfunction.

In adrenaline-induced hypertensive rats, administration of *M. chamomilla* extract at a dose of 40 mg/kg resulted in a significant reduction in blood pressure, with systolic and diastolic values reaching 150 mmHg and 110 mmHg, respectively, by the second hour. These findings suggest that the extract may exert effective blood pressure-regulating activity and holds potential as a candidate for antihypertensive drug development. The findings of our study are consistent with those reported by Awaad et al. (Awaad et al. 2018), in which a single oral administration of *M. chamomilla* extracts (200 mg/kg) led to a reduction in systolic and diastolic blood pressure in normotensive rats at 1, 1.5, and 2 h. The antihypertensive effect of *M. chamomilla* extract was also confirmed (Luo et al. 2020), supporting our findings.

## Conclusions

These results highlight the efficacy of the 40 mg/kg dose of *M. chamomilla* extract in managing blood pressure, making it a promising candidate for further investigations. These findings indicate that *M. chamomilla* extract is a promising agent with the potential to regulate blood pressure. Its ability to both lower and, under certain conditions, raise blood pressure suggests that this extract could be utilized in the development of future antihypertensive and antihypoxic drugs. Molecular docking revealed that the compound apigenin-7-O-neohesperidoside exhibits strong binding affinity to vascular regulatory proteins. Based on the results obtained from the in vitro experiments, it is suggested that the *M. chamomilla* extract affects these ion channels, and this effect is likely attributed to the polyphenol- and flavonoid-rich compounds present in the extract. These compounds are proposed to inhibit ion channels on the cell membrane, particularly Ca²⁺ channels.

## Data Availability

The data used and/or analyzed during the current study are available from the corresponding author on reasonable request.

## Declarations

The manuscript submitted represents our own original work and has not been published and is not being submitted or considered for publication elsewhere. All authors participated in the work in a substantive way and are prepared to take public responsibility for the work. The text, illustrations, and any other materials included in the manuscript do not infringe upon any existing copyright or other rights of anyone. The authors are agreeing that all materials submitted for evaluation (including text, figures, etc.) retains the property/copyrights of The Science of Nature. In the case of not acceptable papers, after their rejection the property of all materials will backward to the authors. Reproduction, posting, transmission or other distribution or use of the article or any material contained therein, requires a citation to the journal The Science of Nature.
